# Cancer Biology of GSPT1: Mechanisms and Targeted Therapy Opportunities of Molecular Glue Degraders

**DOI:** 10.1002/advs.202511789

**Published:** 2025-11-05

**Authors:** Qiqi Lin, Wenjing Liu, Wenjia Lu, Monong Zhao, Lin Cao, Zhiyu Li, Jubo Wang, Xi Xu, Hongxi Wu

**Affiliations:** ^1^ State Key Laboratory of Natural Medicines Department of Pharmacology School of Pharmacy China Pharmaceutical University Nanjing 211198 China; ^2^ School of International Pharmaceutical Business China Pharmaceutical University Nanjing 211198 China; ^3^ State Key Laboratory of Natural Medicines Jiangsu Key Laboratory of Drug Design and Optimization Department of Medicinal Chemistry School of Pharmacy China Pharmaceutical University Nanjing 211198 China

**Keywords:** GSPT1, cancer biological function, molecular glue degrader, precision oncology, translation termination

## Abstract

G1 to S phase transition protein (GSPT1), a small GTPase involved in translation termination, which promotes the progression of cancer cells, has emerged as an attractive potential therapeutic target for cancer treatment with the rapid breakthrough of molecular glue degraders (MGDs). Although the precise mechanism of GSPT1 in cancer biology is partially understood, in this review, the characteristics of GSPT1 expression and regulatory networks are systematically attempted to be addressed, from insights into the structure, expression, and molecular mechanisms, highlighting the distribution and isoform‐specific signaling of GSPT1 in tumors. The clinical significance is emphasized, immune interactions, and oncogenic pathways of GSPT1‐targeted therapies, proposing strategies to address current challenges and provide therapeutic opportunities for the application of GSPT1 degraders in precision oncology. A novel future direction is hoped to provide to enhance the treatment response of GSPT1 MGDs in clinical implications.

## Graphical abstract

This review systemically summarizes the structure, expression, regulatory networks, and isoform‐specific signaling of GSPT1 in cancers. According to the integration of GSPT1 targeted therapies, current challenges of GSPT1 targeted therapies are further analyzed, and provide therapeutic opportunities for the application of GSPT1 degraders in precision oncology.

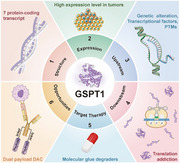

## 1 Introduction

G1 to S phase transition protein (GSPT1), a small GTPase previously “undruggable target” involved in translation termination, has emerged as a potential therapeutic target for the treatment of cancer with the rapid breakthrough of targeted protein degradation (TPD) technology. GSPT1 (also known as the eukaryotic release factor 3a, eRF3a), a critical GTPase, mediates translation termination upon recognition of stop codons (UAA, UAG, UGA)^[^ ^1^, ^2^, ^3^, ^4^, ^5^ ^]^ GSPT1 associates with eRF1 to form the eRF1‐eRF3‐GTP ternary complex, which binds to the elongation ribosome^[^ ^6^, ^7^ ^]^ and stimulates eRF1 activity of releasing nascent peptide chains in a GTP‐dependent manner to promote translation termination when a stop codon enters the A site of the ribosome.^[^ ^8^ ^]^ In addition to its role in translation termination, GSPT1 also participates in ribosome recycling and the nonsense‐mediated mRNA decay (NMD) pathway to avoid cytotoxicity caused by the formation of truncated protein.^[^ ^9^, ^10^ ^]^ Moreover, GSPT1 is involved in cytoskeleton organization and cell cycle regulation in the yeast cell.^[^ ^11^ ^]^ As a multi‐functional protein, GSPT1 critically regulates translation termination, NMD, and cell cycle progression.

Recent studies have revealed that abnormal expression and function dysregulation of GSPT1 are significantly correlated with tumor progression. In 2005, Brito et al. found that the dysfunction of GSPT1 potentially leads to the development of gastric cancer (GC).^[^ ^12^ ^]^ After that, studies recommended that carriers of the longer allele (12‐GGC_n_ glycine codons) in GSPT1 showed a significantly increased tumor risk in GC and breast cancer (BRCA), indicating that GSPT1 has the potential to promote the progression of cancer cells.^[^ ^12^, ^13^ ^]^ Notably, GSPT1 was significantly upregulated in gastrointestinal cancers, including GC, colorectal cancer (CRC), liver cancer, reproduction‐endocrine related tumors such as BRCA, and neuroendocrine‐related tumors, including glioma.^[^ ^14^, ^15^, ^16^, ^17^, ^18^ ^]^ Studies have identified GSPT1 as a prognostic biomarker and promoter of tumors. Knockdown of GSPT1 significantly inhibited tumor proliferation, unlocking the potential therapeutic role of GSPT1 for cancer treatment.^[^ ^19^, ^20^ ^]^ Furthermore, upregulated GSPT1 expression correlates with poor prognosis in triple‐negative breast cancer.^[^ ^21^ ^]^ Nevertheless, as a therapeutic “undruggable target”, the precise mechanism of GSPT1 in cancer biology is still unclear, and the effect of targeting GSPT1 in tumors has not been systematically summarized.

With the rapid development of TPD, which induces the proximity of E3 ubiquitin ligase to the target protein, resulting in ubiquitination and degradation of the target protein in a proteasome‐dependent manner, TPD‐mediated GSPT1 degradation therapy has been an attractive strategy for targeting GSPT1 in tumors.^[^ ^22^, ^23^ ^]^ As one strategy of TPD, Molecular glue degraders (MGDs) that target GSPT1 degradation, such as CC‐885 and CC‐90009 against acute myeloid leukemia (AML)^[^ ^24^, ^25^ ^]^ and MRT‐2359 against MYC‐driven cancers^[^ ^26^ ^]^ have shown promising antitumor activities in preclinical models. Frustratingly, the clinical programs of GSPT1 MGDs have largely encountered failure or major challenges. For example, the clinical trial of BTX‐1188, a dual degrader targeting GSPT1 and IKZF1/3, was halted due to a business decision,^[^ ^27^ ^]^ meanwhile the phase I/II clinical trial of CC‐90009 was terminated due to the lack of efficacy in the short‐term acute phase.^[^ ^28^ ^]^ What we have learned from those clinical trials is that not all patients with different cancer types can respond to GSPT1 degraders, and the mechanisms behind the cancer biology of GSPT1 are still insufficient.

Therefore, a comprehensive and systematic understanding of GSPT1 is urgently needed to provide a pharmacological perspective for targeted therapies in abnormal GSPT1‐driven cancer. In this review, we focused on the current molecular understanding of GSPT1 expression, regulatory networks, and signaling pathways in cancer biology, and summarized the research progress of GSPT1‐targeted therapies. We hope to reveal the therapeutic potential of GSPT1 in precision oncology, providing a novel future direction for enhancing the response to GSPT1‐targeted therapy in clinical trials.

## 2 Characteristics of Human GSPT1

In order to understand the cancer biology of GSPT1 in tumors, we first analyzed the structures, distribution, and function of GSPT1 isoforms and described isoform‐specific functions according to the different domains of GSPT1. Moreover, we also investigated GSPT1 expression patterns, including isoform‐specific expression in pan‐cancer.

### 2.1 Structural Features and Isoforms‐Specific Protein‐Protein Interaction (PPI) Network

**Figure 1:**
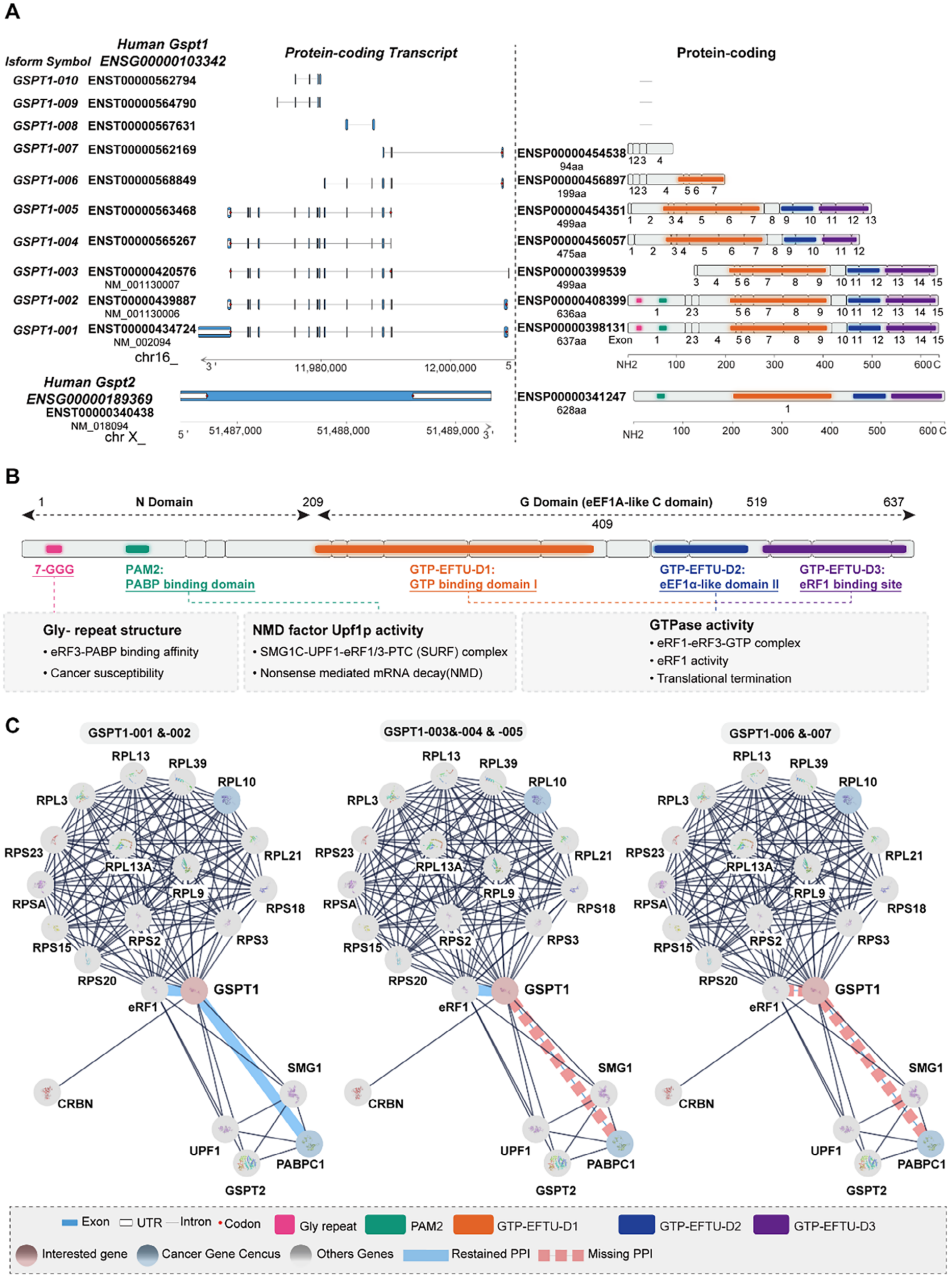
Characteristics of human GSPT1. A) Protein‐coding transcripts and protein structure of GSPT1/2 contain 10 transcripts (isoform symbols GSPT1‐001 to 010). Data were collected, cleaned, and curated from Ensembl, ASCancers Altas, and cBioportal. B) GSPT1 contains five functional domains, each corresponding to a distinct function. C) Top 20 isoform‐specific PPIs Network of GSPT1 based on String Score and ASCancers Altas.

Ensembl database^[^ ^29^ ^]^ has shown that the human GSPT1 gene contains 10 isoforms, 7 of which are protein‐coding transcripts (GSPT1‐001 to GSPT1‐007, **Figure** **1**A). The full length GSPT1 protein (GSPT1‐001, code by NM‐002094) comprises 15 exons coding 637 amino acids, including 1) a non‐conserved N‐terminal domain (N domain), and 2) a canonical guanosine triphosphate (GTP)‐binding domain (G) along with two β‐barrel domains in the C‐terminal domain (C domain), which is homologous to GTP‐binding translation factors, such as EF‐Tu, eEF1A, and the C domain of the ribosome rescue factor Hbs1 (Figure 1B).^[^ ^30^, ^31^ ^]^ The C domain of GSPT1 contains three highly conserved eEF1a‐like domains (GTP‐EFTU‐Domain 1‐3). This region is essential for GTPase activity and eRF1 binding ability to form eRF1‐eRF3‐GTP complex for releasing nascent peptide chains by energy from hydrolysis of GTP to promote the biological process of translation termination.^[^ ^8^ ^]^

Nevertheless, compared with the C domain, the N domain of GSPT1 is more divergent.^[^ ^32^ ^]^ In the N domain, a poly(A)‐binding protein (PABP)‐interacting motif PAM2 is competitively bound by PABPC1 (cytoplasmic isoform of PABP) and UPF1.^[^ ^33^ ^]^ By binding to PABPC1, GSPT1 promotes the translation cycle under the effect of eIF4E and eIF4G.^[^ ^34^ ^]^ Besides, PABPC1 can promote the effect of GSPT1 in translation termination.^[^ ^35^ ^]^ Standard stop codons are typically located in the final exon of mRNA, followed by a short 3′ untranslated region, whereas premature stop codons (PTCs) are associated with a long 3′ untranslated region and the presence of an exon junction complex. PTC increases the separation between the terminating ribosome and the PABP associated with the poly(A) tail, which reduces interaction with GSPT1. Therefore, competitively binding to the GSPT1, NMD factor UPF1 can initiate NMD by the complex of SMG1‐UPF1‐GSPT1‐eRF1 to degrade mRNA with PTCs as a protein quality control mechanism.^[^ ^33^, ^36^, ^37^ ^]^

Only GSPT1‐001 and 002 contain a glycine‐repeat structure (GGC glycine codons, n = 7‐12) in the N domain. Studies reported that the longer allele (12‐GGC glycine codons) reduces the binding affinity between GSPT1 and PABP. Given the role of PABP in translation termination and mRNA degradation, the 12‐GGC allele may inhibit GSPT1‐PABP interaction, thereby impairing the coordination between translation termination and NMD. Besides, 12‐GGC glycine codons increase the cancer susceptibility for BRCA, GC, and CRC.^[^ ^12^, ^13^, ^17^, ^38^, ^39^ ^]^ GSPT1‐002 differs from GSPT1‐001 by only one missed valine in 146 amino acids, while GSPT1‐003 and GSPT1‐005, which are shorter by over two hundred amino acids at the N‐terminus, lack a portion of the 5′ coding region, compared to GSPT1‐001 and GSPT1‐002. Interestingly, NCBI dataset showed that GSPT1‐005 has the same coding DNA sequence as GSPT1‐003 variant encoding the same protein (499 aa, NP‐001123479.1), mainly differing in the 5′ untranslated region.

In addition, analysis of the top 20 isoform‐specific PPIs network of GSPT1 based on the STRING Score revealed that only GSPT1‐001 and 002 possess complete functional domains, with structural basis for involvement in biological processes including NMD and translation termination, while GSPT1‐003, 004, 005, 006, and 007 have missed the PABP and UPF1 binding domain at the N‐domain, which indicates that other isoforms of GSPT1 except for GSPT1‐001 and 002 may lead to dysfunction of NMD (Figure 1C). Furthermore, GSPT1‐006 and 007 have different depletions of the C domain, which is responsible for binding eRF1 and GTPase activity, indicating that isoform‐6 and 7 may lose the function of translation termination. Besides, the isoform‐specific PPIs network showed that all isoforms of GSPT1 interact with various ribosomal protein small subunits and ribosomal protein large subunits, which verified the role of GSPT1 in translation termination. Especially, all isoforms bind to ribosomal protein large subunit 10, an essential structural cancer gene census of the 60S subunit, which mediates ribosome assembly and coordinates translational elongation.^[^ ^40^ ^]^ Moreover, all the isoforms of GSPT1 can interact with cereblon (CRBN), a component of the Cullin‐RING ligase 4‐DDB1‐CRBN‐RBX1 (CRL4^CRBN)^ E3 ubiquitin ligase complex,^[^ ^25^ ^]^ which may indicate that GSPT1 MGDs could degrade all the isoforms of GSPT1 through ubiquitination of E3 ubiquitin ligase complex. Above all, we proposed that the mechanisms of GSPT1 promoting cancer progression may be abnormal expressions of GSPT1 isoforms.

### 2.2 Variations Between GSPT1 and GSPT2

In the human genomes, two distinct genes encoding eRF3 were identified, entitled GSPT1/eRF3a and GSPT2/eRF3b, located on human chromosome 16 and chromosome X, respectively. GSPT2, as the important paralog of GSPT1 sharing 87% homology with GSPT1, also contains one PAM2 motif in the N domain and eEF1A‐like C domain, but mainly differs from human GSPT1 in the N domain, where GSPT2 loses a repetition of GGC glycine codons. GSPT2 may maintain the structural basis of GTPase‐binding and eRF1‐binding activity in the biological process of translation termination^[^ ^32^ ^]^ (Figure 1A). PPI analysis showed that human GSPT2 can interact with UPF1, PABP, eRF1, and GSPT1^[^ ^37^ ^]^ (Figure 1C), indicating that GSPT2 either modulates GSPT1‐mediated biological processes through direct/indirect mechanisms or substitutes for GSPT1. Studies have proved that both GSPT1 and GSPT2 have the ability to bind to eRF1 and promote the release activity of eRF1, which is involved in translation termination.^[^ ^37^, ^41^ ^]^ GSPT1 is the main translation termination factor, while GSPT2, which exhibits low expression levels in mammalian cells, shows the ability to substitute for GSPT1 in the translation termination.^[^ ^2^, ^42^ ^]^ The differences between GSPT1 and GSPT2 are also in tissue distribution and in expression during cell cycle progression at the mRNA level.^[^ ^37^, ^41^ ^]^ As the significant form in most tissues, GSPT1 is highly expressed in all tissues, with the level of GSPT1 changing throughout the cell cycle. Conversely, GSPT2 is expressed mainly in the mouse brain and exhibits low expression in human cell lines according to the data from HPA.^[^ ^37^ ^]^ Nevertheless, the biological role of GSPT2 in GSPT1‐mediated cancers remains unexplored. As GSPT2 is a substitute for GSPT1, the dysregulation of GSPT2 may result in cancer drug resistance to GSPT1‐targeted therapy. Therefore, we proposed that GSPT2‐mediated functional compensation for GSPT1 may be a critical overlooked factor in GSPT1‐targeted therapy, warranting further investigation into the resistance mechanism of GSPT1‐targeted therapy.

### 2.3 Expression Patterns of GSPT1 in Various Tumor Tissues

**Figure 2:**
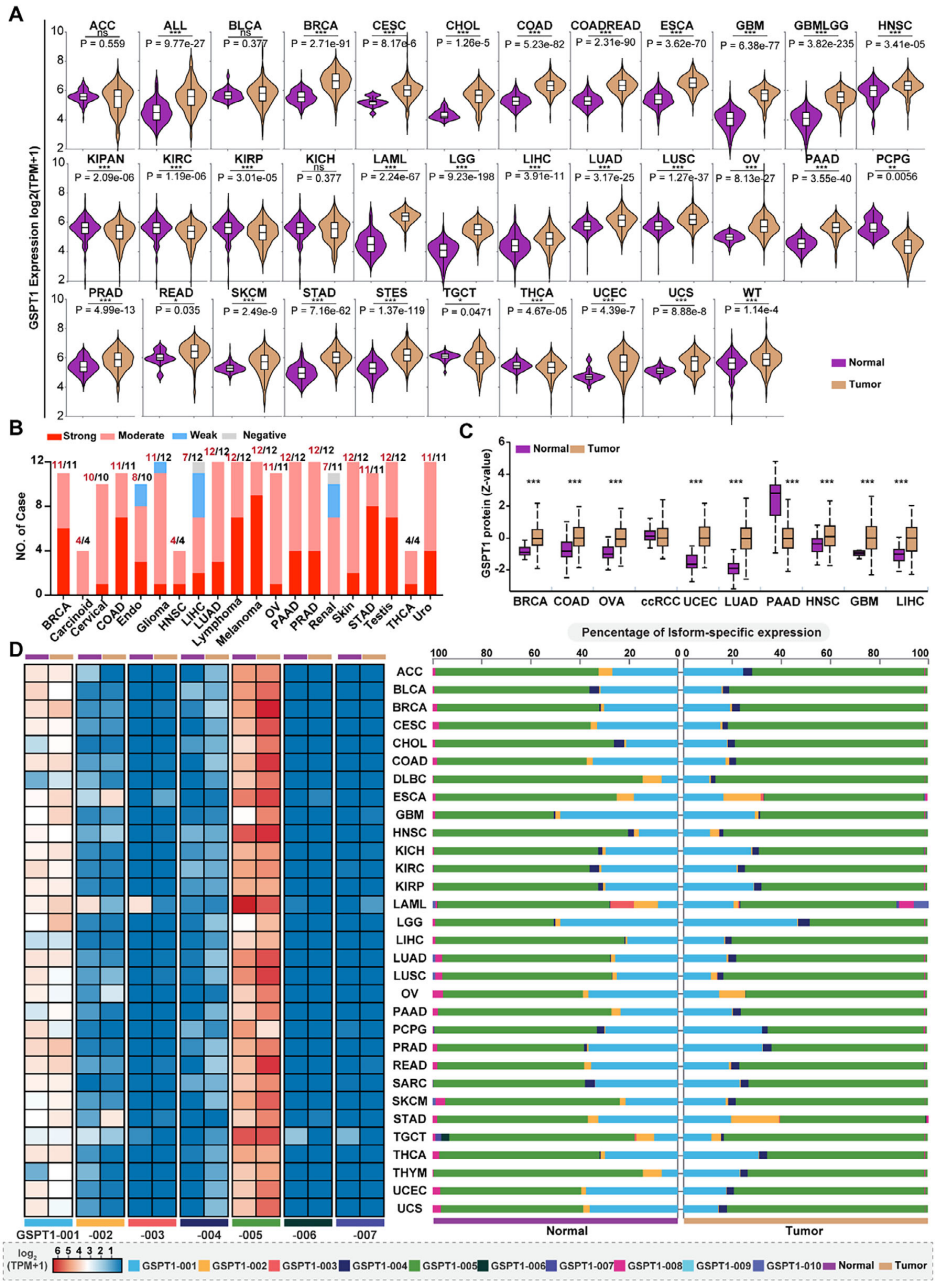
Expression pattern of GSPT1 in tumors. A) mRNA expression of GSPT1 in 34 cancer types derived from UCSC (https://xenabrowser.net/), including TCGA, TARGET, GTEx (PANCAN, N = 19131, G = 60499) datasets. B, C) GSPT1 protein expression in Pan‐cancer including IHC stains of GSPT1 (Antibody HPA052488) in 20 cancer types derived from HPA (https://www.proteinatlas.org/) and CPTAC proteomic expression profile of 10 cancer types derived from UALCAN (https://ualcan.path.uab.edu/analysis‐prot.html). D) Isoform‐specific expression of seven protein‐coding transcripts and percentage of GSPT1 in TCGA Pan‐Cancer using RNA‐seq data derived from GEPIA2 (http://gepia2.cancer‐pku.cn/).

We next summarized the expression of GSPT1 between tumors and normal tissues to discover potential indications that are related to the high expression of GSPT1. Through the pan‐cancer datasets (The Cancer Genome Atlas, TCGA+TARGET+GTEx) downloaded from UCSC,^[^ ^43^ ^]^ we compared the mRNA levels of GSPT1 between tumor tissues and normal tissues across 34 tumors (**Figure** **2**A). Results showed that GSPT1 was upregulated in 73.5% of tumor tissues (25/34) compared to normal tissues. IHC analysis of GSPT1 protein expression derived from HPA indicated that almost all 20‐examined tumor types exhibited strong or moderate GSPT1 stain (Figure 2B). Moreover, the protein expression of GSPT1 between tumor and normal tissues by mass spectrometry analysis derived from CPTAC datasets^[^ ^44^ ^]^ showed that especially highly expressed in BRCA, Colon Adenocarcinoma (COAD), Ovarian Cancer (OV), Uterine Corpus Endometrial Carcinoma (UCEC), Lung Adenocarcinoma (LUAD), Head and Neck Squamous Cell Carcinoma (HNSC), Glioblastoma (GBM), and Liver Hepatocellular Carcinoma (LIHC) compared with the normal tissue (Figure 2C).

We further explored the isoform‐specific expression of GSPT1 in TCGA pan‐cancer derived from RNA‐seq data, to explore whether the correlation between GSPT1 expression and cancer progression is related to different isoforms. The results showed that all seven protein‐coding isoforms of GSPT1 can be detected in tumors, with the highest percentage of GSPT1‐005 expression (up to 75%, log_2_ (TPM+1) ≥5) and second‐highest percentage of full‐length GSPT1‐001 expression (up to 25%) (Figure 2D). Most notably, among the isoforms involved only in translation termination but not in the NMD process, expression of GSPT1‐005 rather than GSPT1‐003 was upregulated in tumors, including BRCA, cervical squamous cell carcinoma, COAD, Esophageal Carcinoma (ESCA), LUAD, lung squamous cell carcinoma, and UCEC tissues when compared to normal tissues. However, expression of the full‐length GSPT1‐001 involved in the two processes was downregulated in tumors. These results indicated that dysregulation of GSPT1 in NMD may increase the abnormal mRNAs expression that leads to the formation of truncated protein, resulting in cancer progression. Besides, the percentage of GSPT1‐002 expression is increased in ESCA tumors but decreased in Stomach Adenocarcinoma (STAD) tumors compared to normal tissues, but the role of this difference has not been revealed in gastrointestinal cancer.

## 3 Upstream Regulatory Mechanisms of GSPT1 in Pan‐cancer

To explicitly clarify the mechanisms underlying the abnormal expression or dysfunction of GSPT1 in cancer, we clarified the upstream regulatory mechanisms, including genomic alterations, transcriptional factors, and microRNA (miRNA), and post‐translational modifications (PTMs).

### 3.1 Genomic Alterations

**Figure 3:**
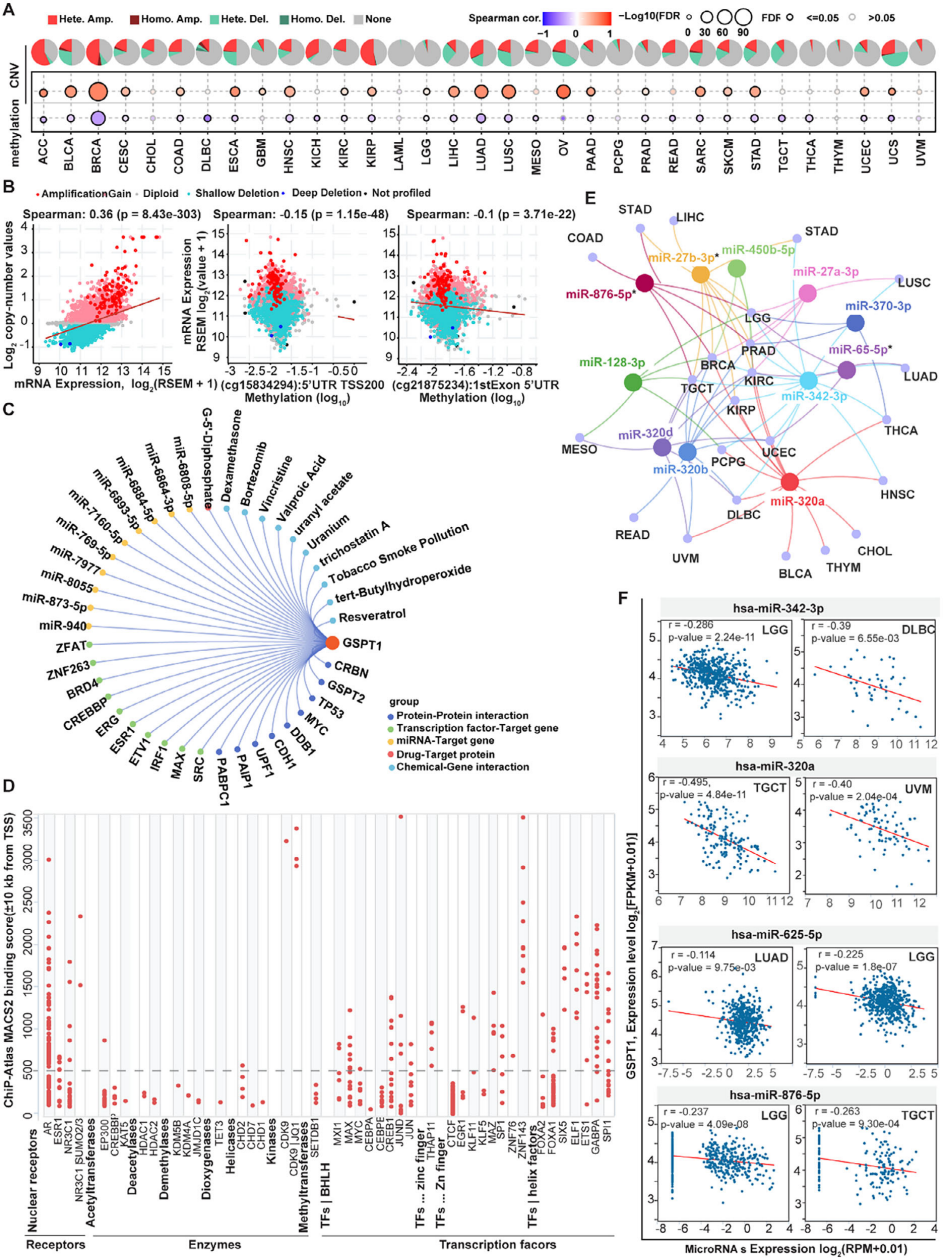
Upstream Regulatory mechanisms that may affect GSPT1/eRF3a expression, including genomic alterations (Mutation, CNV, and DNA methylation), Transcriptional factors, and miRNA. A) Pie plot summarizes the CNV of GSPT1, and bubble chart summarizes the correlation between the CNV or methylation with GSPT1 mRNA expression in each cancer retrieved from GSCALite. B) Correlation between CNV or methylation with GSPT1 mRNA expression in TCGA Pan‐Cancer Altas retrieved from cBioportal. C) Top 10 Regulatory networks based on Protein‐Protein interaction, Transcription factor‐Target gene, miRNA‐Target gene, Drug‐Target protein, Chemical‐Gene interaction were retrieved from the Gendoma web server (https://ai.citexs.com). D) Mining of integrated public ChIP‐Seq (cistromic) datasets to discover relationships between human GSPT1 and genetic and small molecule manipulations of cellular receptors, enzymes, and transcription factors was retrieved from The Signaling Pathways Project.^[^ ^173^ ^]^ E) The miRNA‐GSPT1 regulatory network established by Cytoscape software. Prediction and analysis of upstream miRNAs of GSPT1 in > 3 tumor types retrieved from ENCORI. ^*^ indicated that have been reported and validated in the literature. F) The correlation between the GSPT1 expression with the expression of hsa‐miR‐342‐3p, hsa‐miR‐320a, hsa‐miR‐625‐5p, hsa‐miR‐876‐5p in indicated tumor samples was determined by the starBase database.

Analysis of cBioPortal datasets revealed the human *GSPT1* gene has rare somatic mutations, and *GSPT1* expression is not significantly associated with single‐nucleotide variants across pan‐cancer cohorts (data not shown). However, *GSPT1* mRNA levels showed a strong positive correlation with copy number variations (CNVs), particularly heterozygous amplifications, and a negative association with DNA methylation at the 5′ untranslated region TSS200 and first exon regions (**Figure** **3**A,B). Notably, the negative association between *GSPT1* expression and methylation was consistently observed in multiple malignancies, including BRCA, COAD, GBM, HNSC, LIHC, LUAD, OV, and UCEC. These findings suggested that *GSPT1* upregulation may be driven by CNV‐mediated amplification coupled with hypomethylation of regulatory regions, while the precise molecular mechanisms need to be further investigated.

### 3.2 Transcriptional Regulation Mechanisms of GSPT1 Expression

Results from genomic alterations such as mutations and DNA methylation do not fully explain the significant upregulation of GSPT1 in tumors because hypermethylation is often observed during oncogenesis. Therefore, we further elaborated the potential factors on GSPT1 expression by exploring regulatory networks based on PPI, Transcription factors‐target gene, miRNA‐Target gene, Drug‐Target protein, and Chemical‐Gene interaction (Figure 3C). The PPI analysis showed that proteins including GSPT2, TP53, MYC, DDB1, CDH1, UPF1, PAIP1, and PABPC1 have the ability to interact with GSPT1, among which, both TP53 and MYC have the function of regulating transcription.^[^ ^45^, ^46^ ^]^ The transcription factor‐target analysis identified SRC, MAX, and IRF1 as potential regulators of *GSPT1*, supported by ChIP‐seq evidence. Furthermore, mining of integrated public ChIP‐Seq (cistromic) datasets retrieved from The Signaling Pathways Project (Figure 3D) also confirmed that nuclear receptors (such as AR, NR3C1) showed a relative higher binding score to regulatory region of GSPT1 while enzymes including acetyltransferases (such as EP300, CREBBP, KAT5), deacetylase (such as HDAC1, HDAC2), demethylases (such as KDM5B, KDM4A, JMJD1C), TET3, helicases (such as CHD1) exhibited a relative lower binding score to GSPT1 promoter, indicating that GSPT1 expression may be mainly regulated by transcriptional regulation mechanisms. Notably, more than 25 transcriptional factors including BHLH family (such as MAX/MYC, CREB1, JUN), Zn finger (EGR1, ZNF143) and helix factors (such as FOXA1, ETS1, GABPA) showed a relative higher binding score on GSPT1 promoter, indicating that these transcription factors may be the potential upstream of GSPT1 by regulating transcriptional process of GSPT1 expression. Most importantly, researchers found that MYC promotes transcription of GSPT1, and GSPT1 senses the stop codon of MYC to promote its translation,^[^ ^47^ ^]^ which may reveal the mechanisms driving abnormal expression of GSPT1.

Other transcriptional regulation mechanisms on GSPT1 expression were reported. Studies showed that inhibitor of differentiation protein, a key regulator of both cell cycle and cell differentiation processes, plays a crucial role in facilitating the upregulation of GSPT1 expression by inhibition of NRSF and ZBP89, which are transcriptional co‐repressors.^[^ ^48^ ^]^ However, the precise transcriptional regulation mechanisms of GSPT1 expression in tumors need further investigation.

### 3.3 miRNA Regulation

Studies showed that miRNAs exert anti‐tumor effects by suppressing GSPT1 expression, and long non‐coding RNAs (lncRNAs), which are highly expressed in tumors, could indirectly upregulate GSPT1 expression via downregulating specific miRNAs.^[^ ^49^ ^]^ In non‐small cell lung cancer (NSCLC) cells, lncRNA LINC00511 positively regulates GSPT1 expression by inhibiting miR‐625‐5p to promote proliferation, invasion, and migration.^[^ ^50^ ^]^ LncRNA DLX6‐AS1 was also reported to regulate NSCLC progression by interacting with the miR‐27b‐3p/GSPT1 pathway.^[^ ^49^ ^]^ Besides, miRNA‐508‐3p suppresses the proliferation of human lung cancer cells by directly inhibiting GSPT1 expression.^[^ ^51^ ^]^ In glioma, lncRNA MINCR inhibits the binding of miR‐876‐5p to GSPT1 mRNA, increasing GSPT1 levels and promoting glioma cell proliferation and migration.^[^ ^52^ ^]^ In cervical cancer cells, lncRNA SNHG16 regulates the growth and metastasis of cancer cells by modulating miR‐128, which targets GSPT1.^[^ ^53^ ^]^ GSPT1 is also a direct target of miR‐144 in both CRC and GC.^[^ ^16^, ^54^ ^]^ When the expression of GSPT1 is suppressed by miRNA‐144, cell proliferation regulators such as c‐MYC, survivin, MMP‐28 and Bcl2L15 are down‐regulated.^[^ ^16^ ^]^ In GC cells, miR‐498 and miR‐27b‐3p suppress GSPT1.^[^ ^14^, ^55^ ^]^

In addition, the miRNAs‐Target gene analysis of the top 10 regulatory networks on GSPT1 expression by support of PAR‐CLIP methods revealed that other miRNAs (such as miR‐940 in KSHV‐infected primary effusion lymphoma cell lines^[^ ^56^ ^]^) may regulate the expression of GSPT1 (Figure 3C). We next analyzed the most potent upstream miRNAs affecting GSPT1 expression in pan‐cancers retrieved from ENCORI^[^ ^57^ ^]^ (> in 3 tumor types, Figure 3E). We found among the eight potential miRNAs, 3 miRNAs (such as miR ‐ 27b ‐ 3p, miR ‐ 625 ‐ 5p, miR ‐ 876 ‐ 5p) have been studied as discussed above,^[^ ^50^, ^52^, ^55^ ^]^ another 8 miRNAs (such as miR ‐ 450b ‐ 5p, miR ‐ 27a ‐ 3p, miR ‐ 370 ‐ 3p, miR ‐ 128 ‐ 3p, miR ‐ 342 ‐ 3p, miR ‐ 320a, 320b and 320d) strongly negatively correlated with GSPT1 expression in specific cancers. To delineate tumor‐specific miRNA‐*GSPT1* interactions, we found that the negative correlation between *GSPT* and miR‐342‐3p (in Low‐Grade Glioma, LGG, and Diffuse Large B‐cell Lymphoma, DLBC) or miR‐320a (in Testicular Germ Cell Tumor, TGCT, and Uveal Melanoma, UVM) was significantly stronger (*p* < 0.01) than those observed for miR‐625‐5p (LUAD/LGG) or miR‐876‐5p (LGG/TGCT) (Figure 3F). These results highlighted miR‐342‐3p and miR‐320a as promising candidates for therapeutic targeting of *GSPT1* in LGG, DLBC, TGCT, and UVM. The top 10 drug/chemical‐target gene regulatory networks were also explored, and the results revealed that chemical agents such as dexamethasone, valproic acid, and vincristine can affect GSPT1 expression, but the mechanisms have not been reported.

### 3.4 PTMs

In addition to analyzing genomic and transcriptional mechanisms, we also elucidated the post‐translational modifications of GSPT1.

According to the cbioPortal datasets, GSPT1 is phosphorylated at Gly13, Gly16, Gly20, Met36, Gly46, Ser62, Phe89, Pro98, Ala109, Val120, Val146 and Glu192. Acetylation has occurred at more than 15 residues of GSPT1. And ubiquitination of GSPT1 is at Glu333, Glu490, and Gly627 residues. Besides, GSPT1 has malonylation at Gly108, Pro196, Val213, Lys247, Lys254, Tyr448 and Thr493 and S‐nitrosylation at Thr453 residue.^[^ ^58^ ^]^ In addition to the ubiquitination of GSPT1 that leads to degradation of GSPT1,^[^ ^59^ ^]^ which is induced by MGDs and Proteolysis‐targeting chimeras (PROTACs), no evidence clearly states the function underlying phosphorylation and acetylation of GSPT1. Therefore, according to the universal role of PTMs and the function of GSPT1, we propose the role of PTMs during translation termination, NMD, and cell cycle progression. Ubiquitination may degrade GSPT1 by tagging GSPT1, regulating the activity of GSPT1 in the cell cycle and translation termination. And through phosphorylation, GSPT1 may be activated and engaged in translation termination.^[^ ^60^ ^]^ We estimated that acetylation can regulate the distribution of GSPT1 in cells, thus influencing the role of GSPT1 in translation termination and cell cycle regulation.^[^ ^61^ ^]^ Nevertheless, the precise mechanism underlying PTM of GSPT1 still remains to be understood.

## 4 Physiological Functions of GSPT1

**Figure 4:**
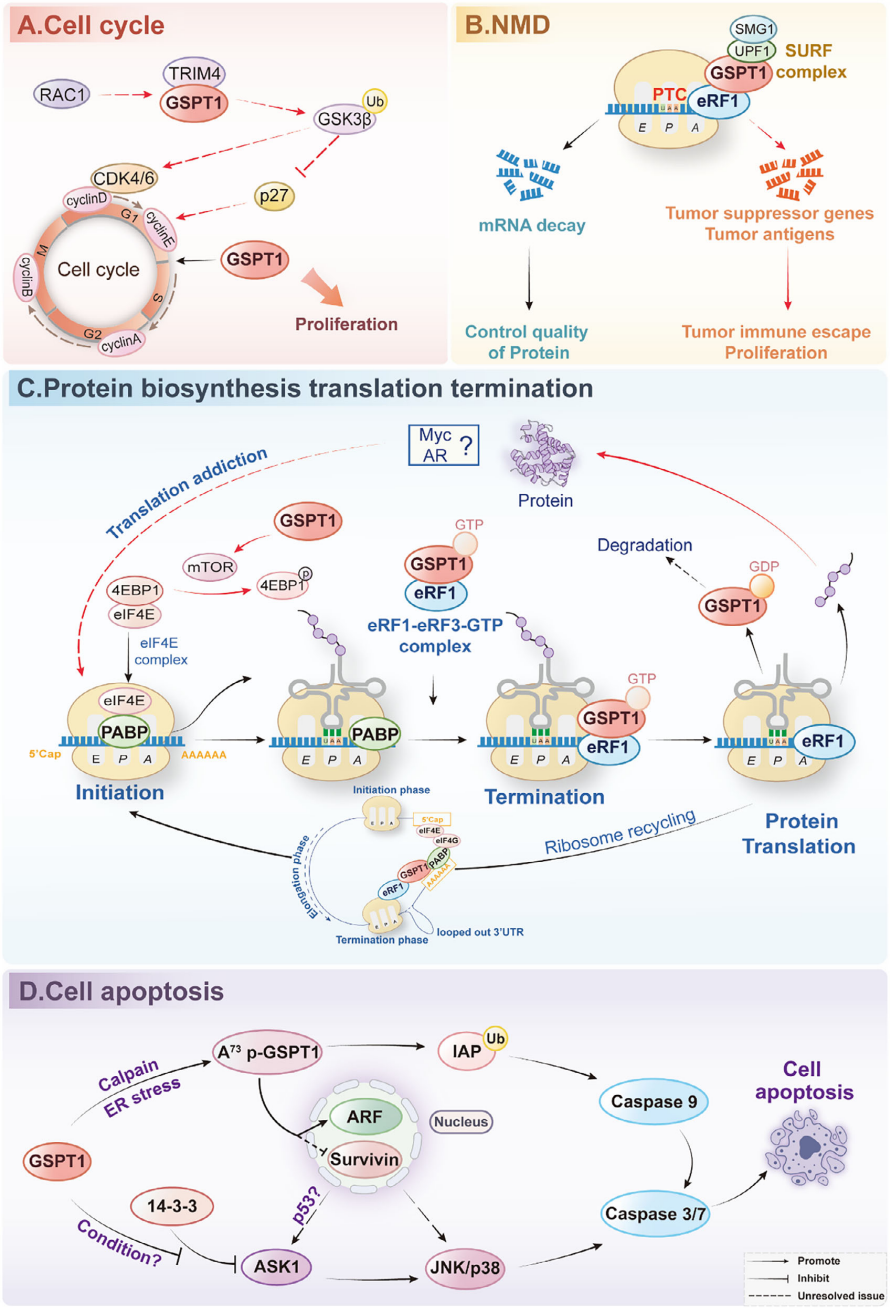
The signaling pathways in cancer and biological functions of GSPT1 in the Cell cycle A), NMD B), Translation termination C), Cell apoptosis D). A) In normal cells, GSPT1 plays an important role in the transition from the G1 to S phase of the cell cycle. In cancer cells, GSPT1 affects the ubiquitination of GSK‐3β by binding to TRIM4, thus regulating the downstream signaling to lead to the inability of the cell cycle to transform to S phase. B) In normal cells, GSPT1 binds to SMG1, UPF1, and eRF1 into a SMG1‐UPF1‐GSPT1‐eRF1 complex, conclusively generating NMD to control the quality of protein. In cancer cells, NMD may degrade the mRNA of tumor suppressor genes and tumor antigens, which promotes tumor immune escape. C) When PABP binds to the stop codons, which recruits ternary compounds (GSPT1, eRF1, and GTP) to the ribosome, GSPT1 participates in peptide release and recycling of post‐termination ribosomes through the activity of GTPase. In cancer cells, upregulated GSPT1 leads to the upregulation of proteins such as MYC by participating in the translation termination process, resulting in tumor translational addiction. D) GSPT1 affects apoptosis regulators under cellular stress conditions, such as ER stress, leading to apoptosis. (Signaling pathways of GSPT1 in tumors are indicated by red arrows, and physiological functions are indicated by black arrows).

GSPT1 is a multi‐functional protein that has crucial effects on translation termination, NMD, and cell cycle regulation. Originally identified as a G1/S‐phase transition regulator in yeast,^[^ ^62^ ^]^ GSPT1 promotes cell cycle progression (**Figure** **4**A). However, how GSPT1 affects the cell cycle remains unknown. We speculated that the function of GSPT1 in G1/S translation of the mitotic cell cycle may be an indirect result of the function of GSPT1 in the process of translation termination and NMD.

### 4.1 NMD

When a PTC occurs, the 3‐poly(A) tail of the PABPC1 connection is further away from PTCs than normal stop codons, resulting in the dysregulation of PABPC1 and competitive association of UPF1 with GSPT1.^[^ ^35^ ^]^ Therefore, GSPT1 can bind to UPF1, SMG1, and eRF1 into SMG1‐UPF1‐GSPT1‐eRF1 complex to initiate NMD (Figure 4B).^[^ ^33^ ^]^ NMD is characterized as a quality control mechanism, which is an important biological process to recognize mRNAs that harbor PTCs, leading to the following degradation of PTCs.^[^ ^63^, ^64^, ^65^ ^]^

### 4.2 Translation Actions

GSPT1 was identified as a translation termination factor to promote translation in cooperation with eRF1 in 1995.^[^ ^1^, ^66^ ^]^ When the A site of the ribosome confronts a stop codon, a pre‐formed ternary complex of eRF1, GSPT1, and GTP is rapidly recruited to the ribosome, which is promoted by PABP.^[^ ^35^, ^67^, ^68^ ^]^ During this period, one function of GSPT1 is to unfasten the structure of eRF1 for stimulating the fast combination with ribosome.^[^ ^69^ ^]^ After that, hydrolyzing GTP facilitates the liberation of GSPT1, GSPT1 thereby rearranges eRF1 into an active structure, which participates in releasing peptide and recycling of post‐termination ribosomes.^[^ ^5^, ^70^ ^]^ Apart from elevating the speed of translation termination, by incorporating an irreversible GTP hydrolysis step between the recognition of the stop codon and the hydrolysis of peptidyl‐tRNA, GSPT1 may also enhance fidelity of translation through kinetic proofreading^[^ ^70^ ^]^ (Figure 4C).

In Cap/Poly(A)‐dependent translation, which is the translational mechanism under normal cellular conditions, GSPT1 binds to PABP via the N domain and interacts with eRF1 through C domain to form an eRF1‐GSPT1‐PABP‐eIF4G complex. This complex connects the terminating ribosome with the 5′ Cap initiation complex, thereby promoting the translation cycle.^[^ ^34^ ^]^

Nevertheless, the post‐translational fate of GSPT1 after translation termination remains obscure; GSPT1 may either participate in subsequent translation termination processes in complex with GDP or undergo ubiquitin‐mediated degradation.

### 4.3 Cell Apoptosis

Under physiological conditions, the full‐length GSPT1 localizes at the endoplasmic reticulum (ER), where GSPT1 functions as an essential translation termination factor. However, under cellular stress conditions such as ER stress, GSPT1 is cleaved at residue A73 mediated by calcium‐dependent calpain. This cleavage event generates the truncated isoform p‐GSPT1, which exposes a previously cryptic inhibitor of apoptosis protein (IAP)‐binding motif at the neo‐N domain. The IAP‐binding motif (AKPF sequence) enables p‐GSPT1 to competitively bind to IAPs, including XIAP, and then promote auto‐ubiquitination and proteasomal degradation of IAPs, preventing inhibitory interaction of IAPs with caspase‐9 and subsequent activating effector caspases‐3/7 to induce apoptosis (Figure 4D).^[^ ^71^, ^72^, ^73^ ^]^

Proteolytic cleavage of GSPT1 not only exposes the IAP‐binding motif but also removes the nuclear export signal located in the N domain. Consequently, p‐GSPT1 acquires nucleocytoplasmic shuttling capability, facilitated by the CRM1‐dependent export pathway. In the nucleus, p‐GSPT1 interacts with key regulatory proteins, including survivin, an IAP family member involved in mitosis and apoptosis inhibition, and p14ARF, which is a well‐characterized tumor suppressor that stabilizes p53 by inhibiting degradation of p53 via MDM2, thereby promoting p53‐dependent apoptosis or cell cycle arrest. However, the precise downstream mechanisms, such as whether p‐GSPT1 influences p14ARF stability, localization, or interaction with p53 regulatory complexes, remain under investigation.^[^ ^74^, ^75^ ^]^ Besides, GSPT1 directly interacts with ASK1, counteracting the inhibitory effect of the ASK1‐binding protein 14‐3‐3. The interaction of GSPT1 and ASK1 promotes ASK1 autophosphorylation and enhances the activation of downstream JNK/p38 MAPK signaling pathways, ultimately leading to apoptosis.^[^ ^76^ ^]^ Studies showed that p53 regulates ASK/JNK/p38 pathway,^[^ ^77^ ^]^ which suggested that p‐GSPT1 may regulate ASK/JNK/p38 pathway through p53 with dual functions.

## 5 GSPT1 Signaling Pathways in Cancer Cells

Despite the emerging role of GSPT1 in oncogenesis, the signaling networks driven by GSPT1 in malignancies remain poorly characterized. The core molecular and physiological function of GSPT1 is the process of protein synthesis and translation termination, which is conserved in both normal and tumor cells. The significant differences of GSPT1 between normal and tumor cells are mainly in the expression level and the resulting biological effects. Therefore, we systematically summarized the signaling pathways of GSPT1 in cancer cells and discussed the molecular and biological role of GSPT1 in cancer in comparison to its physiological functions to promote understanding of the significance of GSPT1 in health and disease. Cleaved GSPT1 also induces cell apoptosis in tumor cells, and signaling pathways may be consistent with those in normal cells (Figure 4D).

### 5.1 Cell Cycle

GSPT1 promotes oncogenic cell cycle progression through multiple mechanisms (Figure 4A). GSPT1 may control the activity of CyclinD1, CDK4, and CDK6 by binding to TRIM4, an E3 ubiquitin ligase, to mediate GSK‐3β degradation, and lead to an indirect impact on CyclinE and CDK2 expression through p21 and p27, which ultimately governs the transition of colon cancer cells from the G1 phase to S phase, facilitating tumor advancement.^[^ ^19^, ^78^ ^]^ However, no in‐depth evidence of GSPT1 on GSK‐3β in cancer through TRIM4 has been revealed. In liver cancer cells, GSPT1 is also positively correlated with high expression of CyclinB1 and CyclinD1.^[^ ^20^ ^]^

### 5.2 NMD

In cancer cells, NMD typically degrades mutant mRNAs of tumor suppressor genes, promoting tumor development. Additionally, NMD prevents the expression of potential tumor antigens, thereby helping tumor cells evade recognition and attack by the immune system and reducing the efficacy of tumor immunotherapy.^[^ ^79^ ^]^ Therefore, in tumor cells, overexpression of GSPT1 may upregulate NMD, which leads to the degradation of tumor suppressor genes and the suppression of the immune system, thereby promoting tumor escape (Figure 4B).

### 5.3 Translation Actions

GSPT1, especially GSPT1‐005, is upregulated in various cancers (Figure 2D), indicating that the upregulated GSPT1 may drive tumor growth through the translation termination process. Furthermore, the potential role of GSPT1 in facilitating peptide chain release and subsequent impact on the synthesis of oncogenic proteins such as MYC and AR remains to be further investigated. Moreover, recent findings have confirmed that the degradation of GSPT1 has remarkable effects on MYC‐driven and AR‐driven solid tumors addicted to protein translation.^[^ ^80^ ^]^

The mammalian TOR (mTOR) pathway plays a critical role in GSPT1‐mediated translation actions. On the one hand, the knockdown of GSPT1 leads to a decrease in the phosphorylation levels of two downstream targets of mTOR, 4E‐BP1 and S6K1, which indicates that GSPT1 may coordinate the balance between translation initiation and termination by regulating mTOR activity.^[^ ^81^ ^]^ On the other hand, studies proved that the depletion of GSPT1 impedes cell cycle progression by inhibiting the mTOR signaling pathway.^[^ ^82^ ^]^ Similar to the role of GSPT1, the overexpression of GSPT2 facilitates the entry of HepG2 cells into the S‐phase through increasing 4E‐BP1 phosphorylation^[^ ^83^ ^]^ (Figure 4C).

### 5.4 Other Proliferative Pathways

GSPT1 knockout resulted in significantly decreased phosphorylation of both ERK and JNK, two key factors of the MAPK signaling pathway, indicating that GSPT1 could potentially enhance cell proliferation, migration, and invasion through the activation of MAPK pathways.^[^ ^20^ ^]^ Furthermore, the upregulation of GSPT1 modulated by Rac1 can be suppressed by JNK, ERK, and NF‐κB inhibitors, indicating that Rac1 upregulates the expression of GSPT1 by JNK, ERK, and NF‐κB, which promotes progression and proliferation of astrocyte cell cycle after central nervous system damage.^[^ ^84^ ^]^ The role of RAC1 and GSPT1 in the cell cycle may be related to the regulation of Cyclin D1 mediated by GSPT1,^[^ ^85^ ^]^ while the mutual relationships between GSPT1 expression and JNK/ERK pathway may be a feedback loop in regulating cell proliferation.

## 6 Degrader Targeting GSPT1 and Combined Therapy Research Progress

**Figure 5:**
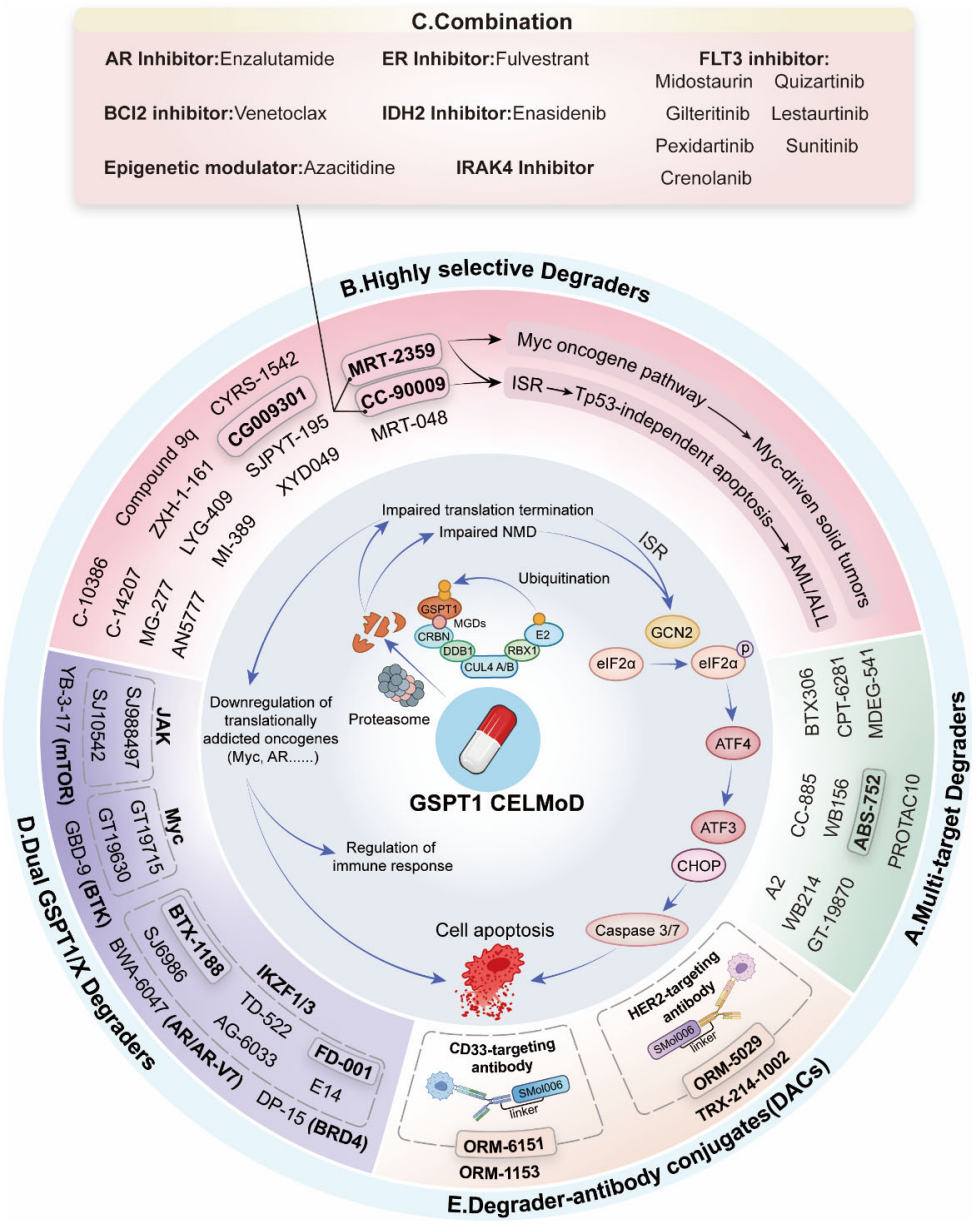
Targeted drug and therapy development of GSPT1 degraders. A) Multi‐target Degraders. B) Highly selective GSPT1 MGDs. C) Combined GSPT1 MGD therapy with target inhibitors. D) Dual target GSPT1/X MGDs. E) Degrader‐antibody conjugates. The dark backdrop represents the investigational drug in the clinical trial.

To date, as a therapeutic “undruggable target”, the majority of pharmacological agents targeting GSPT1 in tumors are GSPT1 degraders. GSPT1 degraders, including PROTACs and MGDs, were identified as CRBN E3 ligase modulators. By recognizing the G‐loop degron of GSPT1, the GSPT1 degrader binds to CRBN and GSPT1 to form a ternary complex, initiating the ubiquitination‐mediated proteasomal degradation of GSPT1.^[^ ^86^, ^87^ ^]^ GSPT1 degradation leads to an increased frequency of stop‐codon readthrough, resulting in increased unfolded peptides and rapid activation of the integrated stress response (ISR) due to damage to GSPT1‐mediated protein translation termination and the NMD process.^[^ ^82^ ^]^ ISR is an evolutionarily conserved signaling network activated by phosphorylating eIF2α. This process is triggered by various internal and external stressors, including amino acid deprivation, oxidative stress, and mitochondrial dysfunction, through the action of the eIF2α kinase activator GCN2 and GCN1.^[^ ^88^, ^89^, ^90^ ^]^ Additional components will be activated to execute cell death if the cellular damage is severe.^[^ ^91^ ^]^ GSPT1 degradation results in the rapid phosphorylation of eIF2a through upregulation of GCN1/2, accumulation of ATF4 and the downstream DDIT4, CHOP, and ATF3, and subsequent cell apoptosis.^[^ ^24^ ^]^ Moreover, downregulation of translational addiction oncogenes (such as MYC and AR) induced by GSPT1 degradation was also observed, ultimately inducing profound anti‐proliferative effects and apoptosis in tumor cells (**Figure** **5**). As discussed in Figure 4D, cleaved‐GSPT1 may induce cell apoptosis through p53 (Figure 4D). Interestingly, degradation induced by GSPT1 MGDs can cause p53‐independent cell death in leukemia while sparing normal hematopoietic stem cells.^[^ ^82^ ^]^

### 6.1 Multi‐Target Degraders

CC‐885, the pioneering GSPT1‐targeting degrader identified from a library of analogs derived from immunomodulatory drug lenalidomide, defined GSPT1 as a CRBN “neosubstrate” specificity on the CRL4^CRBN^ E3 ubiquitin ligase, transforming GSPT1 from an “undruggable” target to an attractive, promising target for the treatment of pan‐cancer (especially for AML). In vitro assay, CC‐885 exhibited sub‐nanomolar potency against 4 out of 5 patient‐derived AML tumor cells and induced Tp53‐independent apoptosis.^[^ ^25^, ^82^ ^]^ However, in subsequent research, CC‐885 was proven as a multiple‐target MGDs by targeting GSPT1 and other CRBN substrates, including IKZF1/3, CK1α, vinculin, BNIP3L, and eRF1, resulting in potent and broad anti‐tumor activity along with severe off‐target toxicity.^[^ ^92^, ^93^ ^]^ According to CDDI data, another multi‐targeted agents targeting GSPT1 and other targets were reported in preclinical development for treatment of cancer, including MDEG‐541 that degrades GSPT1/2, MYC and PLK1,^[^ ^94^ ^]^ WB214 that reduces GSPT1, MDM2 and p53,^[^ ^95^ ^]^ BTX306 that downregulates GSPT1, eRF1, CK1α, MCL‐1 and c‐MYC,^[^ ^96^ ^]^ GT‐19870 that degrades GSPT1, MYC and MYCN,^[^ ^97^ ^]^ and CPT‐6281 that degrades GSPT1, NEK7 and SALL4.^[^ ^98^ ^]^ In addition, PROTAC10 degrades GSPT1, FLT3, and SRC.^[^ ^99^ ^]^ By targeting FLT3, GSPT1, and IKZF1/3, A2 exhibited significantly enhanced antiproliferative activity against drug‐resistant AML cells compared to Gilteritinib (Figure 5A and **Table** **1**).^[^ ^100^ ^]^ The further development of multi‐target degraders might be limited by toxicities. For example, the clinical development of CC‐885 was hampered by significant toxicities associated with various off‐targets, and the potential off‐targets of some multi‐targeted agents were detected.^[^ ^24^, ^94^ ^]^ Studies have revealed that MGDs targeting CRBN can induce degradation of substrates carrying G‐loop degron, a structural degron underlying the pan‐targets of MGDs except for GSPT1. And through urea groups, MGDs, including CC‐885 establish hydrogen bonds with Glu377 of CRBN, contributing to the ternary complex (CRBN‐MGD‐GSPT1) stabilization (**Table** **2**).^[^ ^101^ ^]^

**Table 1:** The development of GSPT1/ERF3a degraders (Clinical stage).

Compound name	Target	Condition	Highest phase	Reference
**Multi‐target prodrugs**				
ABS‐752	NEK7, SALL4, CK1α, CRBN, GSPT1	HCC	Phase I	[102]
**Highly selective MGDs**				
CC‐90009	CRBN, GSPT1	AML, MDS	Phase I/II Terminated NCT04336982	[24]
MRT‐2359	CRBN, GSPT1	Cancer, Solid Tumor	Phase I/II NCT05546268	[80]
CG009301	GSPT1	HMs, AML, ALL, MDS	Phase I CTR20244643	[111]
**Dual target MGDs**				
FD‐001	IKZF1/3, GSPT1	AML, MDS, non‐hodgkin lymphoma (NHL), MM	Phase I/II NCT06731699	[139]
BTX‐1188	IKZF1/3, CRBN, GSPT1	Cancer, Solid Tumor, HMs, AML, Myeloma, MDS, NHL	Phase I Terminated NCT05144334	[135]
**DACs**				
ORM‐6151 (BMS‐986497)	CD33, CRBN, GSPT1	AML, MDS	Phase I NCT06419634	[174]
ORM‐5029 (PTZ‐SMol‐012)	HER2, CRBN, GSPT1	Cancer, Solid Tumor	Phase I NCT05511844	[152]

**Table 2:** The development of multi‐target GSPT1/ERF3a degraders (Preclinical stage).

Compound name	Target	Condition	Reference
Multi‐target MGDs			
BTX306	eRF1, CK1α, MCL‐1, c‐MYC, CRBN, GSPT1	Myeloma	[96]
CC‐885	eRF1, VCL, IKZF1/3, CRBN, GSPT1	Cancer	[175]
GT‐19870	MYC, MYCN, CRBN, GSPT1	Cancer	[176]
CPT‐6281	NEK7, SALL4, CRBN, GSPT1	Cancer, Endocrine Cancer, Neuroendocrine Cancer, Liver Cancer, Lung Cancer	[98]
A2	NEK7, SALL4, CRBN, GSPT1	AML	[100]
**Multi‐target PROTACs**			
MDEG‐541	MYC, PLK1, CRBN, GSPT1/2	GC	[94]
PROTAC10	FLT3, SRC, CRBN, GSPT1	CML, Kidney Cancer	[99]
WB214	MDM2, p53, CRBN, GSPT1	Leukemia	[95]

In addition, ABS‐752 is a prodrug activated by the monoamine oxidase, VAP‐1, to an aldehyde intermediate and subsequently to the active molecule, ABT‐002 has the ability to degrade SALL4, GSPT1, NEK7, and CK1α. VAP‐1, which is overexpressed in cirrhotic liver, was identified as the primary monoamine oxidase responsible for the conversion of ABS‐752. Notably, ABS‐752 is currently in clinical trials for the treatment of HCC (Table 1).^[^ ^102^ ^]^

### 6.2 Highly Selective Degraders

To minimize off‐target toxicity, 15 highly selective MGDs targeting GSPT1 degradation have been developed, 3 of which have entered clinical trials, including CC‐90009, MRT‐2359, and CG009301 (Figure 5B). CC‐90009 (Table 1) represents a selective GSPT1 degrader derived from a structurally optimized derivative of CC‐885, maintaining the potent anti‐AML activity of CC‐885 and selectively degrading GSPT1 to enhance both target specificity and therapeutic safety.^[^ ^103^ ^]^ The anti‐AML activity of CC‐90009 was evaluated on samples from 9 AML patients by the PharmaFlow assay. Viable leukemic cells in 8 of 9 samples decreased rapidly and efficiently at 48 h after treatment, and nearly all were eliminated within 96 h. Studies revealed that GSPT1 degradation induced by CC‐90009 triggered the activation of the integrated stress response pathway, resulting in acute apoptosis.^[^ ^86^, ^104^ ^]^ In the Phase I clinical trial of CC‐90009 for relapsed/refractory (R/R) AML, its toxicity profile was characterized by dose‐limiting toxicities (e.g., hypotension, SIRS, pericarditis with tamponade), common grade 3/4 treatment‐emergent adverse events (e.g., hypocalcemia), along with severe infections and rare treatment‐related deaths, yet the overall toxicity was clinically manageable. Therapeutically, single‐agent efficacy of CC‐90009—manifested as complete remission (CR), morphologic CR with incomplete blood count recovery (CRi), and morphologic leukemia‐free state—was observed in R/R AML patients receiving the Day 1–5 dosing schedule at 3.0 or 3.6 mg.^[^ ^105^ ^]^ Unfortunately, the Phase I/II trial of CC‐90009 in R/R AML or higher‐risk MDS (NCT02848001) was terminated in 2024 due to the lack of efficacy in the short‐term acute phase. The study suggested that the suboptimal pharmacokinetic properties of CC‐90009 may explain limited clinical efficacy.^[^ ^106^ ^]^

MRT‐2359 (Table 1) is a potent, highly selective, and orally bioavailable GSPT1 MGD with nanomolar in vitro IC50 and the most significant clinical potential developed by Monte Rosa Therapeutics. Phase I/II clinical trials of MRT‐2359 have been initiated for the treatment of MYC‐driven solid tumors, including NSCLC, small cell lung cancer, high‐grade neuroendocrine cancer of any primary site, and DLBCL (NCT05546268).^[^ ^80^ ^]^ MRT‐2359 initially degrades GSPT1 by binding to CRBN and the GSPT1 G‐loop degron, which impairs protein synthesis in tumor cells that are addicted to high MYC expression, and indirectly affects MYC expression and MYC‐mediated transcriptional activity (Figure 5B).^[^ ^87^, ^107^ ^]^ MRT‐2359 not only exhibits a strong anti‐proliferative phenotype and broad activity profile in hematological malignancies, including MM, lymphomas, and solid tumors, including PRAD and BRCA, but also possesses a distinct mechanism, exhibiting high sensitivity to MYC‐driven tumors, which are addicted to protein translation, resulting in a dependence on GSPT1.^[^ ^108^ ^]^

However, the clinical trial of MRT‐2359 has encountered setbacks. The trial enrolled volunteers with tumors where high L‐ or N‐MYC expression was expected based on preclinical data. After assessing tissue samples from 46 patients, MYC expression in tissue samples was lower than expected in some tumor types. None of the sampled NSCLC patients had high L‐ or N‐MYC. Although MYC levels were higher in small cell lung cancer, 31%, and high‐grade neuroendocrine tumors, 17%, the expression level of MYC still fell short of expectations. According to preclinical experiments, treatment of MRT‐2359 sensitive prostate cancer cell lines in vitro led to a loss of c‐MYC and, to a lesser extent, AR (including AR‐V7) proteins. In an AR‐positive model with minimal expression of the variant AR‐V7, combinations of MRT‐2359 and enzalutamide were more efficacious than the respective single‐agent treatments, which warrant clinical investigation of MRT‐2359 as a single agent or in combination with an AR antagonist in patients with prostate cancer.^[^ ^109^ ^]^ Therefore, the subsequent clinical trial of MRT‐2359 will focus on prostate cancer.^[^ ^110^ ^]^

CG009301 (Table 1) is a highly selective GSPT1 degrader for the treatment of R/R hematological malignancies developed by Cullgen. The phase I clinical trial of CG009301 for the treatment of Hematological Cancer, AML, ALL, and Myelodysplasia was initiated in December 2024 in China (CTR20244643).^[^ ^111^ ^]^ During the development of CRBN‐based PROTACs targeting the nuclear receptor pregnane X receptor, researchers serendipitously discovered that compound SJPYT‐195 induced pregnane X receptor downregulation through primary degradation of GSPT1, which was further validated by observing similar pregnane X receptor reduction following CC‐885‐mediated GSPT1 degradation.^[^ ^112^ ^]^ Through rational structural optimization, XYD049 is significantly enhanced in selectivity for GSPT1 degradation in castration‐resistant prostate cancer (CRPC), which downregulates CRPC‐related oncogenes in 22Rv1 cells, including AR, AR‐V7, PSA, and c‐MYC.^[^ ^113^ ^]^ In addition, CYRS‐1542 for the treatment of high CRBN expression neuroendocrine cancers,^[^ ^114^, ^115^ ^]^ Compound 9q,^[^ ^116^ ^]^ ZXH‐1‐161,^[^ ^117^ ^]^ LYG‐409,^[^ ^106^ ^]^ MI‐389,^[^ ^118^ ^]^ C‐10386,^[^ ^119^ ^]^ C‐14207,^[^ ^119^ ^]^ MG‐277^[^ ^59^ ^]^ and AN5777,^[^ ^120^ ^]^ MRT‐048^[^ ^110^ ^]^ are identified as highly selective GSPT1 MGDs (**Table** **3**).

**Table 3:** The development of highly selective GSPT1/ERF3a degraders (Preclinical stage).

Compound name	Target	Condition	Reference
**Highly selective MGDs**			
AN5777	CRBN, GSPT1	Leukemia, AML	[120]
compound 9q	CRBN, GSPT1	Leukemia	[116]
CYRS‐1542	CRBN, GSPT1	Neuroendocrine cancers	[114, 115]
C‐10386	CRBN, GSPT1	Cancer, Solid Tumor, AML, Respiratory diseases	[119]
C‐14207	CRBN, GSPT1	Solid Tumor, AML	[119]
LYG‐409	CRBN, GSPT1	Cancer	[106]
MG‐277	CRBN, GSPT1	Breast Cancer, ALL	[59]
MI‐389	CRBN, GSPT1	AML	[118]
MRT‐048	CRBN, GSPT1	Cancer	[177]
ZXH‐1‐161	CRBN, GSPT1	Myeloma	[117]
XYD‐049	CRBN, GSPT1	Cancer, Autoimmune Disease, Inflammation, Viral Infections	[113]
**Highly selective PROTACs**			
SJPYT‐195	CRBN, GSPT1	AML	[112]

### 6.3 Dual GSPT1/X Degraders, Heterobifunctional Degraders, and Combination

The function of GSPT1 in translation termination can be compensated for; thus, the degradation of GSPT1 may lead to drug resistance. By identifying the synthetic lethal partner of GSPT1, researchers hope to identify more sensitive indications for targeting GSPT1, including MYC, IKZF1/3, BTK, AR/ARv7, and mTOR‐driven cancers. Therefore, combination therapy of GSPT1 MGDs with other target therapies, including AR inhibitors, ER inhibitors, BCL2 inhibitors, IDH2 inhibitors, FLT3 inhibitors, and epigenetic modulators, is a novel strategy to overcome drug resistance (Figure 5C). Furthermore, dual GSPT1/X degraders targeting GSPT1 and dual‐mechanism degraders have been developed for the treatment of tumors through the action of two targets, killing two birds with one stone (Figure 5D and **Table** **4**).

**Table 4:** The development of dual target/ Heterobifunctional GSPT1/ERF3a degraders and DACs (Preclinical stage).

Compound name	Target	Condition	Reference
**Dual target MGDs**			
SJ‐6986	IKZF1/3, CRBN, GSPT1	Cancer	[131]
TD‐522	IKZF1/3, CRBN, GSPT1	Myeloma, AML	[138]
AG‐6033	IKZF1/3, CRBN, GSPT1	NSCLC	[137]
E14	IKZF1/3, CRBN, GSPT1/2	Myeloma, Leukemia	[136]
WB156	MDM2, GSPT1	Leukemia	[144]
**Dual target PROTACs**			
DP‐15	BRD4, CRBN, GSPT1	AML	[141]
SJ10542	JAK2/3, CRBN, GSPT1	ALL	[142]
SJ‐988497	JAK, CRBN, GSPT1	Lymphocytic Leukemia	[143]
**Dual target Protein/Nucleic Acids Degrader**			
GT‐19630	MYC, CRBN, GSPT1	Cancer	[128]
GT‐19715	MYC, CRBN, GSPT1	Lymphoma, AML	[127]
**Heterobifunctional degraders**			
GBD‐9	BTK, CRBN, GSPT1	DLBCL, AML	[145]
YB‐3‐17	mTOR, CRBN, GSPT1	Cancer	[149]
BWA‐6047	AR/ARv7, CRBN, GSPT1	Prostate cancer	[147]
**DACs**			
ORM‐1153	GSPT1	Hematologic malignancies	[155]
TRX‐214‐1002	CD33, GSPT1	Leukemia, AML	[156]

#### 6.3.1 Combined Therapy with GSPT1 Degraders

Jointly implementing GSPT1 degraders and target inhibitors on different proteins may overcome resistance and enhance efficacy. Studies revealed that GSPT1 degraders demonstrate superior efficacy when used in combination with ER inhibitors, AR inhibitors, FLT3 inhibitors, BCL2 inhibitors, IDH2 inhibitors, and epigenetic modulators for the treatment of related tumors (Figure 5C). A safety assessment of MRT‐2359 in combination with AR inhibitor enzalutamide for previously treated metastatic prostate cancer, as well as combination with ER inhibitor fulvestrant for previously treated metastatic estrogen receptor‐positive breast cancer, was initiated in 2022 (NCT05546268). Preliminary data indicate that MRT‐2359 combined with enzalutamide demonstrates superior efficacy compared to monotherapy in preclinical models of CRPC and ARv7‐driven prostate cancer. In a clinical trial that three CRPC patients were treated with the combination of MRT‐2359 and enzalutamide, one patient achieved a confirmed partial response (tumor shrinkage of 57%), and the other two patients had stable disease.

FLT3 mutation is the most frequent genetic alteration in AML, with FLT3 internal tandem duplication occurring in ≈25% of adult AML patients and conferring poor overall survival and high rates of treatment resistance development.^[^ ^121^, ^122^ ^]^ FLT3 inhibitors, including sunitinib, pexidartinib, midostaurin, lestaurtinib, crenolanib, and gilteritinib, synergized with CC‐90009 to reduce viability in FLT3‐ internal tandem duplication AML cell lines. Midostaurin enhanced the inhibitory effect of CC‐90009 in primary AML cells, without augmenting the effect of CC‐90009 in CD34^+^ bone marrow mononuclear cells from healthy donors.^[^ ^123^ ^]^

IDH2 mutations occur in 10%–15% of patients with AML. IDH2 exerts a leukemogenic effect through metabolic regulation and leads to a subsequent block in myeloid differentiation.^[^ ^124^ ^]^ The combination of CC‐90009 and mutant IDH2 inhibitor enasidenib enhanced myeloid differentiation and killing of CD34^+^ stem and progenitor cells, and increased differentiated CD235a^+^ erythroblasts. Enasidenib/CC‐90009 combination treatment reduced CD45^+^ malignant populations and increased differentiated CD14^+^ cells, indicating that the combination of CC‐90009 and mutant IDH2 inhibitor can overcome the problem of AML differentiation blockade.^[^ ^123^ ^]^

BCL2 is variably highly expressed in many hematological malignancies, providing protection from cell death induced by oncogenic and external stresses. VEN is the first selective BCL2 inhibitor, as well as the first of a new class of anticancer drugs to be approved for routine clinical practice, currently in chronic lymphocytic leukemia and AML.^[^ ^125^ ^]^ The BCL2 inhibitor VEN enhances the apoptosis and accelerates cell‐autonomous killing induced by CC‐90009 in FLT3‐ internal tandem duplication AML cell lines. In addition, the combination of VEN and CC‐90009 demonstrated enhanced antitumor efficacy in AML patient‐derived bone marrow mononuclear cells, while preserving safety in healthy donor bone marrow mononuclear cells.^[^ ^123^ ^]^

Additionally, the combination of epigenetic modulator azacitidine, which activates tumor suppressor genes through demethylation, and CC‐90009 results in a notable extension of survival.^[^ ^123^ ^]^ However, the evaluation of the combination of CC‐90009 with VEN and azacitidine in a phase I/II trial for patients with AML has been terminated (NCT04336982). Synergies between GSPT1 degraders and other agents may confer significant therapeutic advantages to patients.

To date, researchers have found that CC‐885 has synergistic potential alongside IRAK4 inhibitors. Among CC‐885 substrates, GSPT1 loss showed the most pronounced effects in IRAK4‐inhibited leukemic cells. Transcriptional and proteomic analyses revealed that CC‐885 treatment led to c‐Myc suppression in IRAK4‐deficient leukemic cells. GSPT1 loss reduces translation efficiency, particularly for proteins with short half‐lives, such as c‐Myc. Accelerated c‐Myc protein loss was confirmed following GSPT1 degradation in leukemic cells, with decreased protein stability observed following inhibition of IRAK4, which was validated in AML patient cells, supporting the potential of IRAK4 inhibitors to modulate c‐Myc activity and enhance combinatorial therapies.^[^ ^126^ ^]^

#### 6.3.2 Dual GSPT1/MYC MGDs

MYC‐driven tumors are addicted to protein translation sensed by GSPT1 while MYC promotes transcription of GSPT1, so disruption of the co‐regulatory feedback loop between MYC and GSPT1, through directly targeting MYC/GSPT1 degradation, represents a novel potential approach for MYC‐driven tumors.^[^ ^47^ ^]^ By blocking MYC‐driven transcriptional dysregulation of GSPT1 and GSPT1‐mediated translation of MYC, dual GSPT1/MYC MGDs GT19630 (IC50 in HL‐60 cells was 0.33 nM) and the salt form GT19715 (IC50 in HL‐60 cells was 1.8 nM), exhibited highly active in vivo in multiple therapy‐resistant MYC‐driven tumors. Furthermore, GT19630 and GT19715 are effective against TP53‐mutated and Venetoclax (VEN) ‐resistant cancer models.^[^ ^127^, ^128^ ^]^ GT19630 inhibited cell proliferation, blocked cell cycle progression, promoted apoptosis, and decreased cell migration at low nanomolar concentrations in breast cancer cell lines. Consistent with the ability of MYC to promote immune evasion, GT19630 degraded the negative immune checkpoint inhibitor, B7‐H3.^[^ ^129^ ^]^ Mechanically, GT19630 significantly induced integrated stress response, abrogated oxidative phosphorylation through inhibition of the TCA cycle, and induced cell death.^[^ ^47^ ^]^ Other dual GSPT1/MYC degraders by directly targeting the MYC‐GSPT1 axis were also reported to exhibit powerful antitumor activity in MYC‐driven triple‐negative breast cancer and NSCLC.^[^ ^130^ ^]^

#### 6.3.3 Dual GSPT1/IKZF1/3 MGDs

SJ6986, discovered in the thalidomide analog library, is a potent, selective, and orally bioavailable GSPT1/2 degrader targeting patient‐derived leukemia and medulloblastoma cell lines, while degrading IKZF1/3, with clinical development potential.^[^ ^131^, ^132^ ^]^ SJ6986 effectively reduced the proliferation of DLBCL cells, induced cell apoptosis, and inhibited tumor growth in vivo without significant toxicity. Mechanistically, SJ6986 induces activation of ISR, inhibiting the transition from S to G2/M phase of the cell cycle and triggering oxidative stress and calcium overload in mitochondria and leading to GSPT1‐mediated caspase activation and subsequent cell apoptosis.^[^ ^132^, ^133^, ^134^ ^]^ BTX‐1188 also functions as a dual degrader of GSPT1 and IKZF1/3, with BTX‐1188 serving as an oral molecular glue that exhibits immunomodulatory properties, including the inhibition of pro‐inflammatory cytokines such as IL‐1β, IL‐6, and TNF‐α, as well as IL‐2 production induced by PBMCs activated by LPS and αCD3. In December 2021, BioTheryX, Inc. commenced a Phase I clinical trial (NCT05144334) for BTX‐1188 aimed at treating hematologic and solid tumors. However, the trial was halted on September 18, 2023, due to **a** business decision.^[^ ^27^, ^135^ ^]^ Agents including TD‐522, E14, FD‐001 and AG‐6033, mainly degrade GSPT1, but also reduce the level of IKZF1/3 slightly.^[^ ^136^, ^137^, ^138^, ^139^ ^]^ Phase I clinical trials of FD‐001, a GSPT1/IKZF1/3 degrader, in recurrent/refractory hematologic malignancies, including AML, MDS, NHL, and MM (NCT06731699) were initiated in November 2023 in China.

#### 6.3.4 Dual GSPT1/Other Targets Degraders

BRD4 is an epigenetic regulator implicated in AML progression through transcriptional activation of oncogenes.^[^ ^140^ ^]^ Dual GSPT1/BRD4 degrader DP‐15, exhibited synthetic lethality by dual‐pathway blockade, including transcriptional suppression via BRD4‐MYC axis and translational disruption through GSPT1 degradation. DP‐15 exerts its antiproliferative effects by promoting early apoptosis and inducing G1 phase arrest, thereby inhibiting cell cycle progression in AML and NHL cells.^[^ ^141^ ^]^ Besides, the genetic alterations leading to constitutive JAK‐STAT signaling are driving events for several subtypes of ALL, Dual GSPT1/JAK degraders SJ988497 and SJ10542 displayed potent anti‐tumor activity in JAK‐STAT‐driven cell lines by synthetic lethality through dual mechanisms of impaired transcriptional regulation (JAK‐STAT signaling) and disruption of translational termination (GSPT1 degradation).^[^ ^142^, ^143^ ^]^

WB156 is active in wild‐type and mutant p53‐bearing leukemias due to its ability to degrade both MDM2 and GSPT1 proteins. In cancers that are non‐responsive to MDM2 degradation alone, WB156 acts as a GSPT1 degrader to induce anti‐proliferative effects.^[^ ^144^ ^]^

#### 6.3.5 Heterobifunctional Degraders

By merging PROTAC and MGD for targeting GSPT1 and its synthetic lethal partner concurrently chemically, heterobifunctional degraders are a new design of the double‐mechanism with the characteristics of both MGD and PROTACs. For example, the Bruton Tyrosine Kinase (BTK) protein, which is a tyrosine kinase and a crucial regulator of the BCR pathway, is excessively activated in various lymphoma cells. As the representative GSPT1/BTK heterobifunctional degrader, GBD‐9 efficiently concurrently degraded GSPT1 acting as an MGD and degraded BTK1 acting as a PROTACs by recruiting CRBN, and displayed high activity in various diffuse large B‐cell lymphoma (DLBCL) and AML cell lines. Moreover, GBD‐9 overcomes the limitations of BTK inhibitors and GSPT1 degraders in refractory DLBCL and AML treatment, which may have broader clinical application prospects.^[^ ^145^, ^146^ ^]^

Besides, results from preclinical and clinical trials of MRT‐2359 showed that AR/ARv7 CRPC patients are more sensitive to GSPT1 degraders, indicating that AR/ARv7 is another attractive partner for GSPT1. As another molecule to incorporate MGD into PROTAC degraders, by simultaneously functioning as both MGD targeting GSPT1 and PROTAC degrader targeting AR/ARv7, BWA‐6047 achieves enhanced antitumor effects through blocking androgen signaling and disrupting protein synthesis. Notably, BWA‐6047 maintains high degradation efficiency even in enzalutamide‐resistant cells and remains effective against AR mutants.^[^ ^147^ ^]^

In addition, by integrating the properties of degraders for GSPT1 and inhibitors for its partners together into the same molecule, a dual‐target and dual‐mechanism bifunctional molecule is another design strategy. For example, studies showed that mTOR regulates the initiation of translation primarily by phosphorylating downstream 4EBP1, and mTOR inhibition can enhance the degradation activity of GSPT1 degraders, indicating that combining mTOR inhibition with GSPT1 degradation may overcome resistance and enhance efficacy by disrupting translational processes in tumor cells through dual dual‐target and dual‐mechanism strategy.^[^ ^82^, ^148^ ^]^ Thus, by simultaneously selectively combing the degradation of GSPT1 with robust inhibition of mTOR, YB‐3‐17 not only enhances the original mTOR inhibitory activity, but also improves selectivity while promoting the degradation of GSPT1 MGD, offering a promising direction for precision treatment of tumors with overexpressed mTOR and GSPT1.^[^ ^149^ ^]^

### 6.4 Degrader Antibody Conjugates (DACs)

GSPT1 is ubiquitously expressed in cells; thus, the systematic degradation of GSPT1 may lead to certain side effects. For enhancing targeting specificity, Orum Therapeutics gave us another strategy, which was DACs in 2022. DACs are a novel technology that combine a degrader payload with a monoclonal antibody via a chemical linker, which substitutes a degrader for the payload of ADCs. DAC provides site‐ and tissue‐specific delivery via tumor‐specific and overexpressed antigens that can degrade the target with an improved therapeutic window.^[^ ^150^ ^]^ ORM‐5029 is an innovative, first‐in‐class DAC (Figure 5E). ORM‐5029 (Table 1) consists of SMol006, a highly effective degrader of GSPT1, linked to pertuzumab, which is a human epidermal growth factor receptor (HER2) antibody that has been clinically validated. ORM‐5029 demonstrated strong in vitro and in vivo efficacy across various HER2‐expressing models, showing comparable effectiveness to trastuzumab deruxtecan and notable activity in models resistant to trastuzumab emtansine. Preclinical data demonstrated that ORM‐5029 exhibited 10‐ to 1000‐fold enhanced efficacy in HER2‐expressing cell lines compared to treatment with SMol006, ADC targeting HER2 (Kadcyla and Enhertu).^[^ ^151^, ^152^ ^]^ Cytotoxic activity of ORM‐5029 in tumor xenograft models significantly surpassed SMol006, CC‐885, as well as Kadcyla and Enhertu.^[^ ^153^ ^]^ Phase I trial of ORM‐5029 for treating HER2‐expressing solid tumors was started on 3 October 2022 (NCT05511844).^[^ ^152^ ^]^

Besides, through optimization of linker‐payloads in medicinal chemistry, Orum Therapeutics developed another DAC ORM‐6151 (Table 1), which linked SMol006 to OR000283 (a CD33‐targeting antibody) via an innovative β‐glucuronide releasable linker. ORM‐6151 treatment in CD33‐expressing cell lines showed picomolar activity with 10‐1000‐fold greater potency compared to several GSPT1 degrader molecules, including CC‐90009 and ADC targeting CD33, Mylotarg. ORM‐6151 also exhibited picomolar potency in in vitro cytotoxicity to primary R/R AML patient blasts, with better potency than CC‐90009 and Mylotarg. Moreover, ORM‐6151 showed minimal cytotoxic activity to healthy hematopoietic progenitor cells, with 10‐10000‐fold less toxicity than CC‐90009 or Mylotarg.^[^ ^154^ ^]^ Phase I clinical trial of ORM‐6151 in participants with R/R AML or MDS started on 5 May, 2024 (NCT06419634). However, the clinical data of ORM‐5029 and ORM‐6151 remain undisclosed.

Nevertheless, according to the internal assessment of the clinical progress and the previously disclosed information of ORM‐5029, the clinical development of ORM‐5029 has been terminated on April 28, 2025. Meanwhile, a new agent ORM‐1153, for the treatment of hematological malignancies has been nominated. ORM‐1153 (Table 4) is a novel DAC drug developed by combining the catalytic mechanism of dual‐precision TPD technology with the tumor‐targeting ability of therapeutic antibodies. ORM‐1153 targets GSPT1 and an undisclosed tumor‐associated antigen to enhance selectivity and broaden the therapeutic window. In both in vitro and in vivo experiments, ORM‐1153 demonstrated significant anti‐tumor effects and safety.^[^ ^155^ ^]^

In addition, researchers developed a novel DAC, TRX‐214‐1002 (Table 4), that was comprised of a linker, a monoclonal antibody (Mylotarg), and a novel proprietary GSPT1 MGD, targeting CD33 and GSPT1. TRX‐214‐1002 exhibited the significant ability to degrade GSPT1 and enhanced antileukemic activity, particularly in TP53‐mutated and VEN‐resistant AML cell lines, along with significant antitumor activity superior to ORM‐6151.^[^ ^156^ ^]^

### 6.5 Other Targeting Therapeutic Approaches

Currently, researchers have utilized siRNA and CRISPR/Cas9 system‐mediated knockdown of GSPT1 in experimental models.^[^ ^20^ ^]^ Additionally, targeting specific miRNAs, including miR‐342‐3p and miR‐320a, has been shown to indirectly downregulate GSPT1 expression, thereby demonstrating considerable therapeutic potential.

## 7 Challenges and Opportunities in the Future Directions

From the obtained bioinformatic results and the collected information, GSPT1 is a biologically compelling and highly promising anti‐cancer target. However, to date, GSPT1‐targeting strategies have consistently encountered clinical setbacks. The development of MRT‐2359 for MYC‐driven NSCLC was converted to the development of prostate cancer due to the inability to determine MYC expression levels of patients, while the HER2‐DAC ORM‐5029 pipeline was also discontinued. Therefore, we analyzed challenges encountered by GSPT1 degraders and provided suggestions to realistically avoid these pitfalls in the future and improve this research field.

### 7.1 Application of CRBN‐Humanized Mice in Preclinical Experiments

Researchers have found that there are species differences between human and murine models CRBN, which may result in the lack of comprehensiveness in preclinical data.^[^ ^157^ ^]^ For example, preclinical CC‐885 exhibited multi‐organ toxicity in CRBN humanized murine models, but no toxicity was observed in wild‐type mice.^[^ ^158^ ^]^ Therefore, in the future, researchers can utilize CRBN‐humanized murine models to provide more reliable results for preclinical experiments of agents.

### 7.2 Precision Oncology

As a result of ubiquitous expression of GSPT1 in both normal and cancer tissues, the GSPT1 MGDs had the problem of a narrow therapeutic window due to systemic degradation, which was summarized as “on target, off cancer toxicity”. Therefore, to achieve precise treatment, uncovering indications for tumor cells that are highly dependent on GSPT1 is a potential strategy.

**Figure 6:**
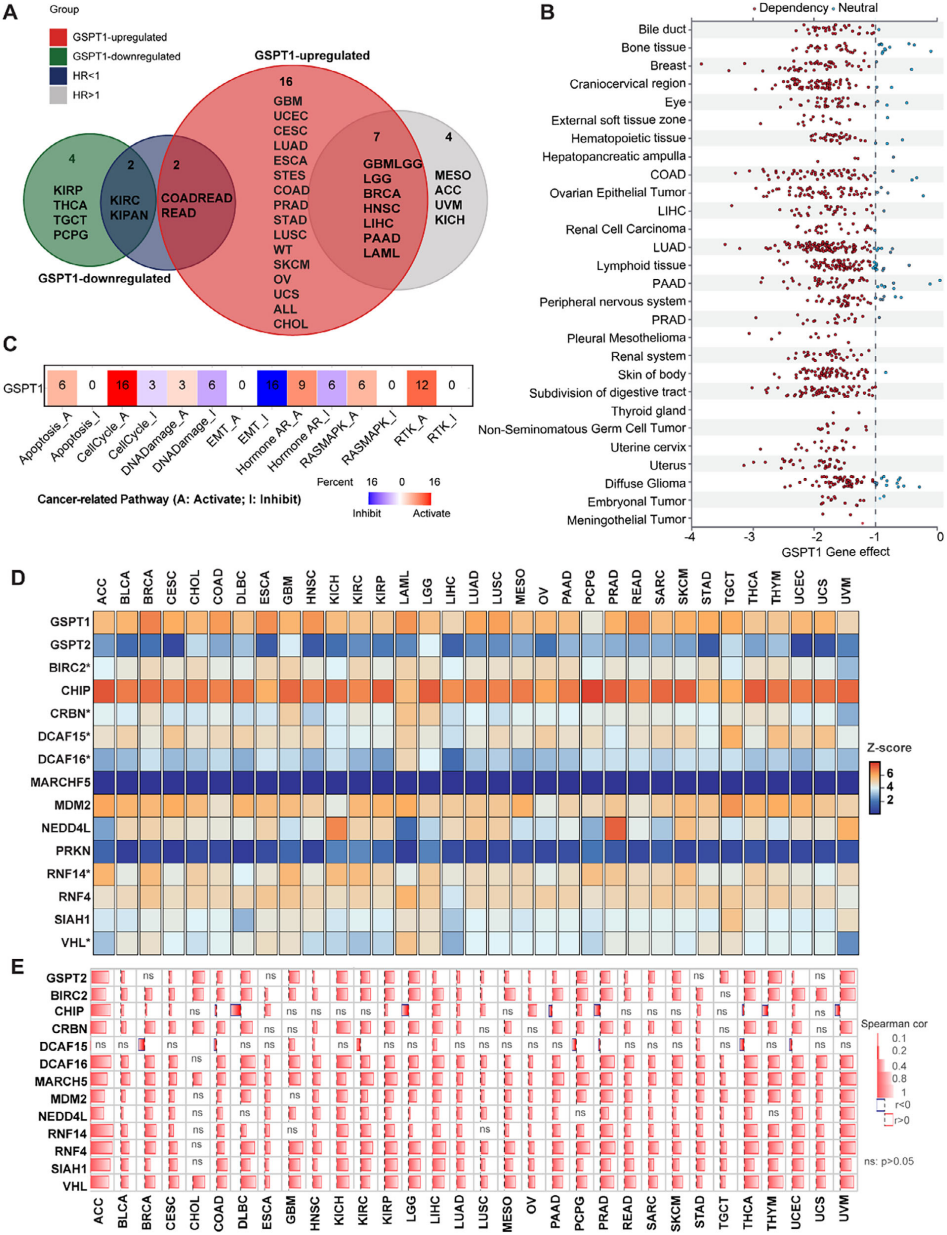
Clinical significance of abnormal GSPT1 expression on prognostic signature of pan‐cancer and function in tumor cells and cancer‐related pathway. A) The prognostic signature of abnormal eRF3a expression in Pan‐cancer derived from UCSC (https://xenabrowser.net/). B) Gene essentiality assessment for GSPT1 obtained through CRISPR loss‐of‐function screens in a wide range of cancer cell lines derived from Cancer DepMap Portal (https://depmap.org/portal/). Gene effect<‐1 indicated dependency. C) Heatmap summarizes the percentage of cancers in which GSPT1's mRNA expression has a potential effect (FDR < = 0.05) on 10 famous cancer‐related pathway activities (TSC/mTOR, RTK, RAS/MAPK, PI3K/AKT, Hormone ER, Hormone AR, EMT, DNA Damage Response, Cell Cycle, and Apoptosis pathways), among 32 cancer types derived from GSCA. The number in each cell indicates the percentage. A: activated; I: inhibited. The expression and pathway activity module estimates the difference in gene expression between pathway activity groups (activation and inhibition), which are defined by median pathway scores. When the pathway activity score in GSPT1^High expression^ tumors > pathway activity score in GSPT1^low expression^ tumors, we consider GSPT1 may have an activating effect on a pathway; otherwise, it has an inhibitory effect on a pathway. D) Association of GSPT1 expression and E3 ligase expression in Pan‐cancer, * indicates that the PPI analysis revealed an association between the E3 ligase and GSPT1. E) Heatmap displaying the Log_2_ fold‐change of gene expression level of GSPT1 and E3 ligase according to the TCGA GEPIA database.

We explored the associations between the abnormal GSPT1 expression patterns and overall survival, disease‐specific survival, disease‐free interval, and progression‐free interval in the Pan‐Cancer Atlas (Table S1). GSPT1 is upregulated in GBM, UCEC, cervical squamous cell carcinoma, LUAD, ESCA, STES, COAD, PRAD, STAD, lung squamous cell carcinoma, WT, SKCM, OV, UCS, ALL, CHOL, GBMLGG, LGG, BRCA, HNSC, LIHC, PAAD, and LAML (**Figure** **6**A and Table S2). Among the tumors in which GSPT1 is upregulated, we found that the expression of GSPT1 positively correlated with poor prognosis in GBMLGG, LGG, BRCA, HNSC, LIHC, PAAD, and LAML (HR > 1).

What's more, apart from degrader ‐antibody conjugates (DACs) that can achieve the “on‐target, on‐cancer”, the development of X‐drug Conjugates, which consist of targeted carriers, chemical linker, and cytotoxic payload, including antibody‐drug conjugates (ADCs), especially dual payload ADCs, radionuclide drug conjugates^[^ ^159^ ^]^ and peptide‐drug conjugates^[^ ^160^ ^]^ will play a pivotal role in alleviating the current predicament. For example, dual payload ADCs link two different payloads (targeting synergistic targets) to the targeting antibody through chemical linkers to achieve synergistic efficacy or overcome drug resistance.^[^ ^161^ ^]^ By combining the synthetic lethal partner of GSPT1, which was discussed in 5.3 Dual GSPT1/X degraders, Heterobifunctional degraders and Combination, and the GSPT1 degrader, dual payload ADC might be a new strategy for overcoming the current setbacks. What's more, the development of prodrugs/prodegraders could also overcome the problem underlying MGDs of “on target, off cancer toxicity”.^[^ ^102^ ^]^

### 7.3 Cancer‐related Pathway

In preclinical exploration of MRT‐2359, researchers found that treatment of MRT‐2359 sensitive prostate cancer cell lines in vitro led to a loss of c‐MYC and, to a lesser extent, AR (including AR‐V7) proteins. This experience indicated that exploring signaling pathways associated with GSPT1 may promote the realization of precision treatment. Although gene essentiality assessment for GSPT1 through CRISPR loss‐of‐function screens in a wide range of cancer cell lines derived from Cancer DepMap Portal verified that almost all cancer cells are dependent on GSPT1 effects^[^ ^162^ ^]^ (Figure 6B), the precise mechanism of GSPT1 modulation remains to be elucidated. To uncover the potential cancer biology of GSPT1‐mediated oncogenesis, we explored the correlation between GSPT1 expression and the activity of 10 well‐known cancer‐related pathways (Figure 6C). The expression of GSPT1 has a significantly positive correlation with the activation of the cell cycle and RTK, while negatively associated with the inhibition of EMT. In addition, the expression of GSPT1 is positively associated with the activation of apoptosis, DNA damage, AR, and RAS‐MAPK pathway. Research on how these cancer‐related pathways interact with GSPT1 in cancer biology may be one future topic.

### 7.4 Refinement of MGDs that Target Different E3 Ligases

Targeting CRBN is currently the most important strategy for GSPT1 MGDs. The availability of the agent to CRBN determines whether the drug will work. Deletion of ILF2 or ILF3 blocks the maturation of full‐length CRBN mRNA, reducing the response to CC‐90009.^[^ ^24^ ^]^ Therefore, the expression of mRNA and protein of CRBN in patients will affect the effect of agents on patients. What's more, cancer cells with high CRBN expression exhibited marked sensitivity to GSPT1 MGDs compared to other cancer types.^[^ ^115^, ^163^ ^]^ However, the challenges of GSPT1 MGDs clinical trials may imply that targeting different E3 ligases will promote the development of more potent GSPT1 MGDs by enhancing degradation efficiency and overcoming potential resistance mechanisms. Furthermore, according to Spearman correlation between GSPT1 and the 10 most frequently utilized E3 ligases, the expression levels of CRBN in various cancer types are not prominent; thus, we predicted the possibility of developing MGDs targeting other E3 ligases in the future (Figure 6D,E).

E3 ligases can be classified into four types: HECT type, U‐box type, RBR type, and RING‐finger type, according to the differentiation of structure and function. NEDD4L is a member of HECT type, while CHIP is from U‐box type, and PRKN is a member of the RBR family.^[^ ^164^ ^]^ RING E3 ligases are divided into monomeric RING finger and multi‐subunit E3 ligases. Monomeric RING E3 ligases not only have the domain for substrate binding and ubiquitination, but also have the function of autoubiquitination, such as MDM2. Multi‐subunit E3 ligases, including CRLs, such as DCAF15 and DCAF16 are a highly diverse class of ubiquitin ligases characterized by several common features.^[^ ^165^ ^]^ RNF4, SIAH1, and RNF14 are members of the RING family.^[^ ^166^ ^]^ Targeting E3 ligases, including VHL and CRBN, members of CRL2 and CRL4, has been developed for the treatment of R/R diseases.^[^ ^94^, ^167^ ^]^

We found that VHL is highly expressed and is highly correlated with GSPT1 in BRCA, GBM, LGG, and LHC. And String PPI analysis showed that GSPT1 is physical associated with VHL.^[^ ^168^ ^]^ The results indicated that developing GSPT1 MGDs targeting VHL in BRCA, GBM, LGG, and LHC may be a promising strategy. Besides, recent studies have revealed that the CRL3 family, traditionally considered challenging to be reprogrammed,^[^ ^169^ ^]^ can be effectively harnessed by MGs for TPD, which exhibits the potential of MGDs targeting other E3 ligases except for CRBN and VHL. CHIP was highly expressed in most cancer types, and MDM2, RNF14, and RNF4 were expressed in most tumors. In addition, in most cancer types, except for CHIP and DCAF15, other ligases are highly associated with GSPT1. CHIP has a high correlation with GSPT1 in ACC, and DCAF15 has a correlation with GSPT1, which may give suggestions to the design of GSPT1 MGDs. In addition, we also suggest developing GSPT1 MGDs targeting BIRC2 in CHOL, LGG, MESO, PRAD, THCA, and THYM, targeting MDM2 in ACC, PRAD, and UYM, NEDD4L in UYM, RNF14 in ACC, KICH, KIRC, KIRP, PRAD, THCA, THYM, and UVM, RNF4 in GBM, THCA, and THYM, SIAH1 in LGG.

### 7.5 The Immunosuppressive Function of GSPT1

According to the clinical data of CC‐90009, a dose‐dependent decrease in GSPT1 levels in T cells was observed, with a >90% decrease observed for higher dose levels, which indicated that targeting GSPT1 may affect the function of immune cells.^[^ ^105^ ^]^ The single‐cell type specificity derived from the HPA shows that GSPT1 RNA expression is mainly expressed on blood & immune cells, and the single‐cell type expression cluster is mainly enriched in T‐cells and monocytes.^[^ ^170^ ^]^ Therefore, we explored the relationship between immune cells and the expression of GSPT1.

**Figure 7:**
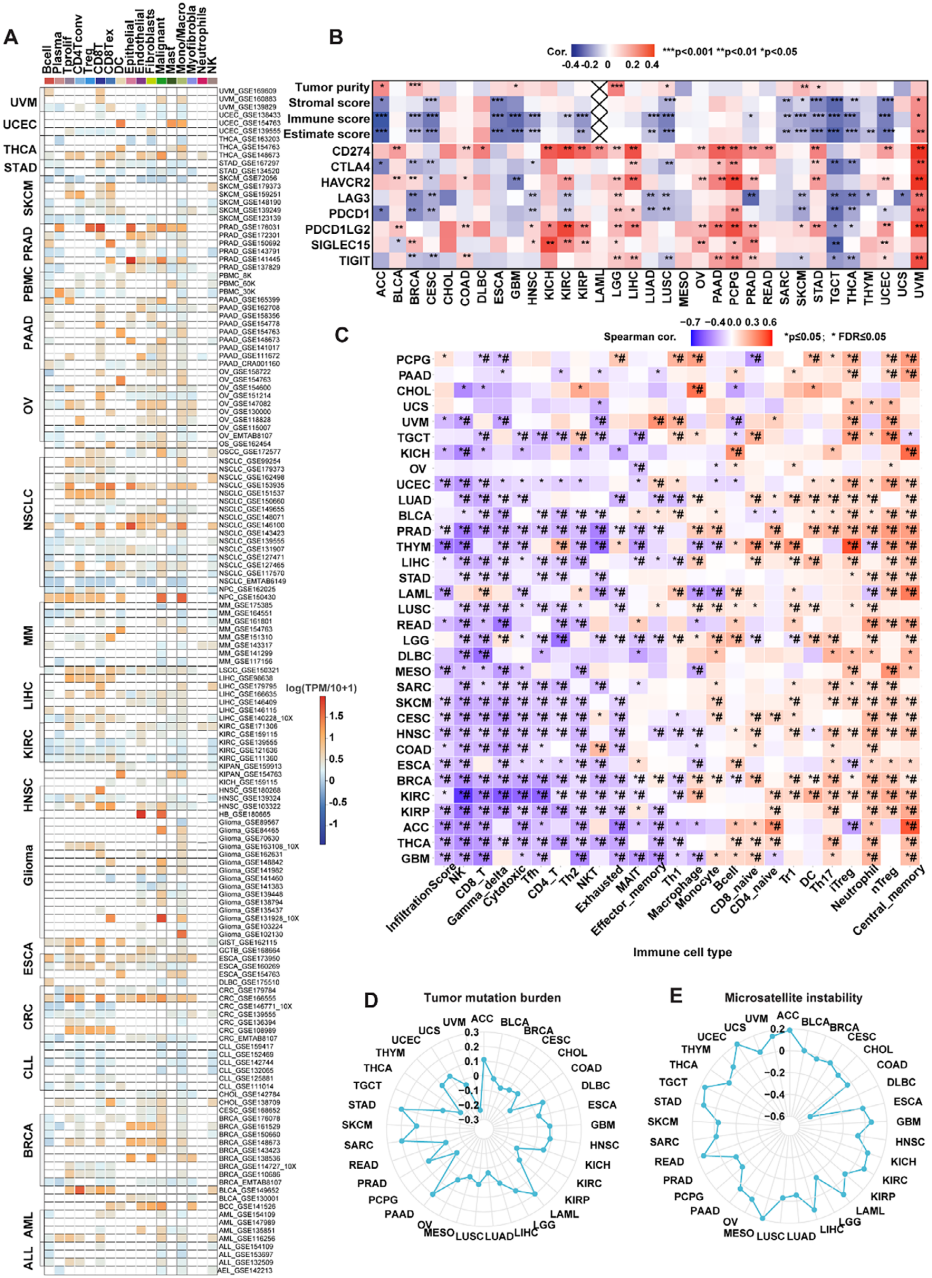
Association between GSPT1 expression in immune cells and immune infiltration. A) Heatmap of single‐cell GSPT1 mRNA expression in major‐lineage pan‐cancer retrieved from TISCH based on scRNA‐seq. B) Heatmap summarizes the Spearman correlation analysis of GSPT1 expression with tumor purity, stromal score, immune score, the ESTIMATE score, and the association between GSPT1 expression with ICB expression: positive correlation in red, negative correlation in blue. ^*^ *p* < 0.05, ^**^ *p* < 0.01, ^***^ *p* < 0.001. C) Spearman correlation analysis of gene set variation analysis (GSVA) score and immune cell infiltration. ^*^ *p* < 0.05; #FDR < 0.05. D,E) Spearman correlation analysis of GSPT1 with TMB and microsatellite instability in pan‐cancer.

According to a heatmap of single‐cell GSPT1 mRNA expression in major‐lineage pan‐cancer retrieved from TISCH based on scRNA‐seq, GSPT1 is highly expressed in immune cells, particularly in Treg cells and CD8 T cells, which infiltrate the cells of PRAD, THCA, NSCLC, LIHC, HNSC, ESCA, and CRC (**Figure** **7**A). Through single‐cell RNA‐seq analyses in pan‐cancer, we explored the correlation between GSPT1 expression and immune infiltration of immune cells in tumors by evaluating the correlation between GSPT1 with tumor purity, stromal score, and immune score (Figure 7B). The results showed that the expression of GSPT1 is positively correlated with tumor purity, while it is negatively correlated with stromal score and immune score, which indicates that GSPT1 may have a role in tumor microenvironment remodeling. Besides, we investigated immune checkpoint proteins, including CD274, CTLA4, HAVCR2, LAG3, PDCD1, PDCD1LG2, and TIGIT, which put the brakes on immune cell functions to regulate immune activation, but the activity of immune checkpoint proteins is exploited in tumors to evade immune surveillance and attack.^[^ ^171^ ^]^ The results showed that CD274, HAVCR2, PDCD1LG2, and SIGLEC15 are positively related to GSPT1, while LAG3 and PDCD are negatively associated with GSPT1 in most cancers. What's more, GSPT1 is positively related to all immune checkpoint proteins in KICH, LGG, OV, PAAD, PCPG, STAD, and UVM.

Thus, pan‐cancer immune infiltration analysis was performed to further explore the correlation between the expression of GSPT1 and tumor microenvironment remodeling (Figure 7C). The results showed that GSPT1 is negatively correlated with the infiltration of NK, CD8 T, γδ T, CD4 T, and Th2, but is positively correlated with central memory and neutrophil cells. In summary, the results suggested that the expression of GSPT1 may be correlated with immunosuppressive activity. Through Spearman correlation analysis of GSPT1 with tumor mutation burden (TMB) and microsatellite instability in pan‐cancer (Figure 7D,E), we found that GSPT1 is significantly positively associated with the TMB of SARC, STAD, PAAD, and LAML and the microsatellite instability of ACC, UCS, TGCT, READ, lung squamous cell carcinoma, LIHC, LAML, KIRC, KICH, and GBM. In conclusion, dysregulation of GSPT1 expression may promote tumorigenesis through immunosuppressive effects.

Considering the immunosuppressive effect of GSPT1, the combination of GSPT1 degraders and immunotherapy, including PD‐1 inhibitors, may inhibit tumor growth, activate immune attacks, disrupt immune escape, and enhance anti‐cancer efficacy.

## 8 Conclusion and Perspectives

As a previously undruggable target, GSPT1 plays significant roles in translation termination, NMD, and the cell cycle, which has been a captivating topic with the rapid breakthrough of TPD.^[^ ^172^ ^]^ Therefore, a deeper comprehension of the physiological function underlying multiple GSPT1 mechanisms and associated signaling pathways would allow for more precise manipulation of GSPT1 to improve cancer therapy. In this review, we attempt to offer scientific evidence to enhance the understanding of GSPT1 expression, regulatory networks, and signaling functions in cancer progression, revealing potential therapeutic targets and contributing to the pharmacological development of GSPT1‐targeted agents and therapy.

In addition to describing the structures of different GSPT1 isoforms, we also offer a deeper understanding of the functions and expressions of GSPT1 isoforms. We found that the upregulation of GSPT1‐005 may be the mechanism underlying the abnormal expression of GSPT1 that drives the tumor's progression. Thus, we explored the upstream regulatory mechanisms, including genomic alterations (mutations, single‐nucleotide variants, CNV, and DNA methylation), transcriptional factors, and miRNAs. The results showed that, except for CNV and miRNA, transcriptional factor ID1 may lead to the abnormal expression of GSPT1. Moreover, we also predicted that receptors, including AR, ESR1, and NR3C1, enzymes, including EP300, CDK9, and CHD2, and more than 25 transcriptional factors may contribute to the dysfunction of GSPT1.

We summarized the downstream mechanisms of GSPT1 and provided an overview of the advancements and challenges of degraders targeting GSPT1 in preclinical research and clinical trials. From the obtained bioinformatic results and the collected information, GSPT1 is a biologically compelling and highly promising anti‐cancer target. However, to date, GSPT1‐targeting strategies have consistently encountered clinical setbacks. We analyzed the underlying mechanisms and provided suggestions, including the Application of CRBN‐humanized mice in preclinical experiments, precision oncology, cancer‐related pathways, refinement of MGDs that target different E3 ligases, and the immunosuppressive functions of GSPT1.

In summary, GSPT1 MGD therapeutic approaches represent a highly promising direction. Although some setbacks have emerged at present, by taking measures to address the aforementioned issues, the current difficulties can be effectively surmounted, thereby facilitating and accelerating the clinical progress of GSPT1 MGDs. We hope to provide potential indications and precise therapeutic strategies for targeting GSPT1, and ultimately to improve potential clinical cancer therapy in the near future.

## 9 Experimental Section

### Acquisition of Characteristics of Human GSPT1

Ten protein‐coding transcripts and protein structures of GSPT1 were retrieved from public databases, including Ensembl (https://www.ensembl.org/index.html?redirect=no), Genecards (https://www.genecards.org/), UniProtKB (https://www.uniprot.org/), NGDC databases (https://www.cncb.ac.cn/services?lang=en), the ASCancers Atlas (https://ngdc.cncb.ac.cn/ascancer/home), and cBioportal (https://www.cbioportal.org/). In addition, we explored the top 20 isoform‐specific PPI networks for GSPT1 by the String Score (https://cn.string‐db.org/) and ASCancers Atlas.

### Expression Pattern of GSPT1 in Tumors

Based on multi‐omics data platforms, we analyzed GSPT1 expression patterns across pan‐cancer. Transcriptomic data from the UCSC Xena platform (https://xenabrowser.net/)—integrating TCGA, TARGET, and GTEx datasets (covering 34 cancer types and 19131 samples)—revealed differential mRNA expression of GSPT1 in multiple cancers. At the protein level, immunohistochemistry results from the Human Protein Atlas database (https://www.proteinatlas.org/) demonstrated significant heterogeneity of GSPT1 expression across 20 cancer tissue types, while proteomic data from CPTAC (https://pdc.cancer.gov/pdc/) and UALCAN (https://ualcan.path.uab.edu/analysis‐prot.html) further validated GSPT1 protein expression characteristics in 10 cancer types. In addition, analysis of TCGA pan‐cancer RNA‐seq data using the GEPIA2 platform (http://gepia2.cancer‐pku.cn/) uncovered the specific expression profiles and relative abundance of seven protein‐coding transcripts of GSPT1, with all gene expression levels normalized to transcripts per million.

### Correlation Analysis of Genomic Variations (Mutations, Single Nucleotide Variations, Copy Number Variations, and DNA Methylation), Transcription Factors, and miRNAs With GSPT1/eRF3a

We systematically investigated the regulatory mechanisms of GSPT1 through multi‐dimensional genomic analyses. Using GSCALite (https://guolab.wchscu.cn/GSCA/#/), we summarized the CNV landscape of GSPT1 and evaluated the correlations between CNV/DNA methylation and GSPT1 mRNA expression across various cancers. To explore the regulatory network of GSPT1, we retrieved the top 10 regulatory interactions—including protein‐protein, transcription factor‐target gene, miRNA‐target gene, drug‐target protein, and chemical‐gene interactions—from the Gendoma web server (https://ai.citexs.com). Additionally, integrated analysis of public ChIP‐Seq datasets from the Signaling Pathways Project (http://www.signalingpathways.org/index.jsf) and the OpenTargets platform (https://platform.opentargets.org/) revealed relationships between GSPT1 and various cellular receptors, enzymes, and transcription factors. For miRNA‐mediated regulation, we constructed a miRNA‐GSPT1 regulatory network using Cytoscape based on ENCORI (https://rnasysu.com/encori/) predicted upstream miRNAs conserved across more than three tumor types, with literature‐validated miRNAs marked accordingly. Finally, the starBase database (https://rnasysu.com/encori/) analysis confirmed the correlation between GSPT1 expression and specific miRNAs (hsa‐miR‐342‐3p, hsa‐miR‐320a, hsa‐miR‐625‐5p, hsa‐miR‐876‐5p) in corresponding tumor samples.

### The Prognostic Signature of Abnormal eRF3a Expression

Data was collected from UCSC pan‐cancer (https://xenabrowser.net/) and PrognoScan (https://dna00.bio.kyutech.ac.jp/PrognoScan/index.html).

### Gene Essentiality Assessment for GSPT1

Gene essentiality of GSPT1 was evaluated using genome‐scale CRISPR‐Cas9 loss‐of‐function screening data obtained from Cancer DepMap Portal (https://depmap.org/portal/).

### The Relationship Between GSPT1's mRNA Expression and 10 Famous Cancer‐Related Pathways

The regulatory effects of GSPT1 on cancer‐related pathways were analyzed using data obtained from the Gene Set Cancer Analysis (GSCA) platform (https://guolab.wchscu.cn/GSCA/#/). This comprehensive analysis encompassed 32 cancer types and focused on 10 major oncogenic pathways: TSC/mTOR, RTK, RAS/MAPK, PI3K/AKT, Hormone ER, Hormone AR, EMT, DNA Damage Response, Cell Cycle, and Apoptosis. Pathway activity scores were calculated based on the median expression of core pathway genes. The regulatory direction of GSPT1 was determined by comparing pathway activity score between GSPT1‐high and GSPT1‐low expression groups, with statistical significance set at FDR ≤ 0.05.

### Association of GSPT1 Expression and E3 Ligase Expression in Pan‐cancer

The relationship between GSPT1 and E3 ligase expression was analyzed using data from two public databases. Gene expression patterns were obtained from the TCGA GEPIA database (http://gepia.cancer‐pku.cn/), while correlation analyses were performed using the Timer2 database (http://timer.cistrome.org/).

### Association between GSPT1 Expression in Immune Cells and Immune Infiltration

The pan‐cancer single‐cell expression profile of GSPT1 was obtained from the TISCH database (http://tisch.comp‐genomics.org/). Spearman correlation analyses were subsequently performed to assess the associations of GSPT1 expression with tumor microenvironment scores (including tumor purity, stromal, immune, and ESTIMATE scores), immune checkpoint gene expression, immune cell infiltration levels (based on GSVA scores), TMB, and microsatellite instability.

### Research Resource Identifiers (RRIDs)

Our study did not utilize any cell lines; therefore, Research Resource Identifiers (RRIDs) and contamination checks for cell lines are not applicable.

## Conflict of Interest

The authors declare no conflict of interest.

## Author Contributions

Q.L. performed the investigation, figure, formal analysis, wrote the original draft, and wrote, reviewed, and edited. W.L. performed data curation and formal analysis, writing the original draft. W.L. performed the investigation, figure, formal analysis, and wrote the original draft. M.Z. and L.C. wrote the original draft. Z.L. oversaw and provided insights. J.W. and X.X. performed funding acquisition, writing, reviewing, and editing. H.W. performed conceptualization, funding acquisition, investigation, data curation and formal analysis, supervision, writing the original draft and writing, reviewing, and editing.

## Consent for Publication

All authors have consented to submit this article for publication.

## Supporting information



Supporting Information

## References

[1] G. Zhouravleva , L. Frolova , X. Le Goff , R. Le Guellec , S. Inge‐Vechtomov , L. Kisselev , M. Philippe , EMBO J. 1995, 14, 4065. 7664746 10.1002/j.1460-2075.1995.tb00078.x PMC394485

[2] C. Chauvin , S. Salhi , C. L. Goff , W. Viranaicken , D. Diop , O. Jean‐Jean , Mol. Cell. Biol. 2005, 25, 5801. 15987998 10.1128/MCB.25.14.5801-5811.2005 PMC1168810

[3] Z. Cheng , K. Saito , A. V. Pisarev , M. Wada , V. P. Pisareva , T. V. Pestova , M. Gajda , A. Round , C. Kong , M. Lim , Y. Nakamura , D. I. Svergun , K. Ito , H. Song , Genes Dev. 2009, 23, 1106. 19417105 10.1101/gad.1770109 PMC2682955

[4] S. Hoshino , H. Miyazawa , T. Enomoto , F. Hanaoka , Y. Kikuchi , A. Kikuchi , M. Ui , EMBO J. 1989, 8, 3807. 2511002 10.1002/j.1460-2075.1989.tb08558.x PMC402068

[5] S. Shao , J. Murray , A. Brown , J. Taunton , V. Ramakrishnan , R. S. Hegde , Cell 2016, 167, 1229. 27863242 10.1016/j.cell.2016.10.046 PMC5119991

[6] L. Chavatte , L. Frolova , L. Kisselev , A. Favre , Eur. J. Biochem. 2001, 268, 2896. 11358506 10.1046/j.1432-1327.2001.02177.x

[7] L. Frolova , X. Le Goff , H. H. Rasmussen , S. Cheperegin , G. Drugeon , M. Kress , I. Arman , A.‐L. Haenni , J. E. Celis , M. Phllippe , J. Justesen , L. Kisselev , Nature 1994, 372, 701. 7990965 10.1038/372701a0

[8] L. Frolova , X. L. Goff , G. Zhouravleva , E. Davydova , M. Philippe , L. Kisselev , RNA 1996, 2, 334. 8634914 PMC1369376

[9] L. E. Maquat , J. Cell Sci. 2005, 118, 1773. 15860725 10.1242/jcs.01701

[10] S. Hoshino , Wiley Interdiscip. Rev. RNA 2012, 3, 743. 22965901 10.1002/wrna.1133

[11] I. A. Valouev , V. V. Kushnirov , M. D. Ter‐Avanesyan , Cell Motil. Cytoskeleton 2002, 52, 161. 12112144 10.1002/cm.10040

[12] M. Brito , J. Malta‐Vacas , B. Carmona , C. Aires , P. Costa , A. P. Martins , S. Ramos , A. R. Conde , C. Monteiro , Carcinogenesis 2005, 26, 2046. 15987717 10.1093/carcin/bgi168

[13] M. Miri , S. Hemati , F. Safari , M. Tavassoli , Med. Oncol. 2012, 29, 1581. 22101789 10.1007/s12032-011-0111-x

[14] R. Liu , X. Han , S. Gao , Y. Chen , J. Zhang , J. Clin. Lab. Anal. 2023, 37, 24810. 10.1002/jcla.24810 PMC9937881 36597856

[15] Y.‐Q. Xi , L.‐H. Xu , L.‐J. Yang , H.‐Q. Wang , T.‐C. Yang , Z. Li , W. Xie , J.‐W. Zhang , X.‐F. Li , M.‐H. Feng , Curr. Med. Sci. 2022, 42, 100. 34985612 10.1007/s11596-021-2463-6

[16] R. Xiao , C. Li , B. Chai , Biomed. Pharmacother. 2015, 74, 138. 26349975 10.1016/j.biopha.2015.08.006

[17] J. Malta‐Vacas , C. Chauvin , L. Gonçalves , A. Nazaré , C. Carvalho , C. Monteiro , D. Bagrel , O. Jean‐Jean , M. Brito , Oncol. Rep. 2009, 21, 1551. 19424636 10.3892/or_00000387

[18] T. Sasayama , T. Hamada , K. Tanaka , H. Nagashima , S. Yamanishi , T. Ueyama , Cell Death Dis. 2024, 15, 572. 39117611 10.1038/s41419-024-06967-1 PMC11310507

[19] X. Long , L. Zhao , G. Li , Z. Wang , Z. Deng , Aging 2021, 13, 10354. 33819920 10.18632/aging.202796 PMC8064227

[20] Y.‐Q. Xi , J.‐B. Gao , X.‐F. Li , L.‐H. Xu , Z. Li , L.‐J. Yang , J. Wang , H.‐Q. Wang , X.‐C. Fang , S.‐R. Huang , W. Xie , M.‐H. Feng , J.‐W. Zhang , Curr. Med. Sci. 2023, 43, 104. 36459303 10.1007/s11596-022-2665-6

[21] S. Wang , A. Beeghly‐Fadiel , Q. Cai , H. Cai , X. Guo , L. Shi , J. Wu , F. Ye , Q. Qiu , Y. Zheng , W. Zheng , P.‐P. Bao , X.‐O. Shu , Breast Cancer Res. Treat. 2018, 171, 199. 29748761 10.1007/s10549-018-4816-9 PMC7195858

[22] G. M. Burslem , C. M. Crews , Cell 2020, 181, 102. 31955850 10.1016/j.cell.2019.11.031 PMC7319047

[23] E. S. Toriki , J. W. Papatzimas , K. Nishikawa , D. Dovala , A. O. Frank , M. J. Hesse , D. Dankova , J.‐G. Song , M. Bruce‐Smythe , H. Struble , F. J. Garcia , S. M. Brittain , A. C. Kile , L. M. McGregor , J. M. McKenna , J. A. Tallarico , M. Schirle , D. K. Nomura , ACS Cent. Sci. 2023, 9, 915. 37252349 10.1021/acscentsci.2c01317 PMC10214506

[24] C. Surka , L. Jin , N. Mbong , C.‐C. Lu , I. S. Jang , E. Rychak , D. Mendy , T. Clayton , E. Tindall , C. Hsu , C. Fontanillo , E. Tran , A. Contreras , S. W. K. Ng , M. Matyskiela , K. Wang , P. Chamberlain , B. Cathers , J. Carmichael , J. Hansen , J. C. Y. Wang , M. D. Minden , J. Fan , D. W. Pierce , M. Pourdehnad , M. Rolfe , A. Lopez‐Girona , J. E. Dick , G. Lu , Blood 2021, 137, 661. 33197925 10.1182/blood.2020008676 PMC8215192

[25] M. E. Matyskiela , G. Lu , T. Ito , B. Pagarigan , C.‐C. Lu , K. Miller , W. Fang , N.‐Y. Wang , D. Nguyen , J. Houston , G. Carmel , T. Tran , M. Riley , L. Nosaka , G. C. Lander , S. Gaidarova , S. Xu , A. L. Ruchelman , H. Handa , J. Carmichael , T. O. Daniel , B. E. Cathers , A. Lopez‐Girona , P. P. Chamberlain , Nature 2016, 535, 252. 27338790 10.1038/nature18611

[26] O. B. Wallace , G. Gavory , M. Ghandi , A.‐C. d'Alessandro , D. Bonenfant , M. Cabanski , L. Cantagallo , A. Chicas , Q. Chen , A. Diesslin , C. King , V. Massafra , R. Narayan , A. Osmont , D. Peck , C. P. Ortiz , M. Schillo , A. Singh , R. Tiedt , S. Tortoioli , S. Buonamici , M. Warmuth , F. Janku , B. Fasching , Cancer Res. 83, ND10.

[27] I. Biotheryx , An Open Label, Escalating Multiple Dose Study to Evaluate the Safety, Toxicity, Pharmacokinetics, and Preliminary Activity of BTX‐1188 in Subjects With Advanced Malignancies clinicaltrials.gov 2023 (accessed: November 2025).

[28] Celgene, A Phase 1, Open‐label, Dose Finding Study of CC‐90009, a Novel Cereblon E3 Ligase Modulating Drug, in Subjects With Relapsed or Refractory Acute Myeloid Leukemia or Relapsed or Refractory Higher‐Risk Myelodysplastic Syndromes clinicaltrials.gov 2025 (accessed: November 2025).

[29] Ensembl genome browser, https://www.ensembl.org/index.html?redirect=no (accessed: November 2025).

[30] C. Kong , K. Ito , M. A. Walsh , M. Wada , Y. Liu , S. Kumar , D. Barford , Y. Nakamura , H. Song , Mol. Cell 2004, 14, 233. 15099522 10.1016/s1097-2765(04)00206-0

[31] A. M. G. van den Elzen , J. Henri , N. Lazar , M. E. Gas , D. Durand , F. Lacroute , M. Nicaise , H. van Tilbeurgh , B. Séraphin , M. Graille , Nat. Struct. Mol. Biol. 2010, 17, 1446. 21102444 10.1038/nsmb.1963

[32] G. Zhouravleva , V. Schepachev , A. Petrova , O. Tarasov , S. Inge‐Vechtomov , IUBMB Life 2006, 58, 199. 16754297 10.1080/15216540600686862

[33] P. V. Ivanov , N. H. Gehring , J. B. Kunz , M. W. Hentze , A. E. Kulozik , EMBO J. 2008, 27, 736. 18256688 10.1038/emboj.2008.17 PMC2265754

[34] N. Uchida , S. Hoshino , H. Imataka , N. Sonenberg , T. Katada , J. Biol. Chem. 2002, 277, 50286. 12381739 10.1074/jbc.M203029200

[35] A. Ivanov , T. Mikhailova , B. Eliseev , L. Yeramala , E. Sokolova , D. Susorov , A. Shuvalov , C. Schaffitzel , E. Alkalaeva , Nucleic Acids Res. 2016, 44, 7766. 27418677 10.1093/nar/gkw635 PMC5027505

[36] G. Kozlov , K. Gehring , PLoS One 2010, 5, 10169. 10.1371/journal.pone.0010169 PMC2854688 20418951

[37] S. Hoshino , M. Imai , M. Mizutani , Y. Kikuchi , F. Hanaoka , M. Ui , T. Katada , J. Biol. Chem. 1998, 273, 22254. 9712840 10.1074/jbc.273.35.22254

[38] S. Jerbi , B. Jolles , T. Bouceba , O. Jean‐Jean , RNA Biol. 2016, 13, 306. 26818177 10.1080/15476286.2015.1137421 PMC4829321

[39] J. Malta‐Vacas , P. Ferreira , C. Monteiro , M. Brito , Cancer Genet. Cytogenet. 2009, 195, 132. 19963113 10.1016/j.cancergencyto.2009.08.010

[40] D. Pollutri , M. Penzo , Cells 2020, 9, 2503. 33227977 10.3390/cells9112503 PMC7699173

[41] C. G. Jakobsen , T. M. Segaard , O. Jean‐Jean , L. Frolova , J. Justesen , Mol. Biol. 2001, 35, 575. 11524954

[42] C. Le Goff , O. Zemlyanko , S. Moskalenko , N. Berkova , S. Inge‐Vechtomov , M. Philippe , G. Zhouravleva , Genes Cells 2002, 7, 1043. 12354098 10.1046/j.1365-2443.2002.00585.x

[43] UCSC Genome Browser Home, https://genome.ucsc.edu/ (accessed: November 2025).

[44] CPTAC | Office of Cancer Clinical Proteomics Research, https://gdc.cancer.gov/about‐gdc/contributed‐genomic‐data‐cancer‐research/clinical‐proteomic‐tumor‐analysis‐consortium‐cptac (accessed: November 2025).

[45] C. Vaughan , I. Pearsall , A. Yeudall , S. P. Deb , S. Deb , Subcell Biochem. 2014, 85, 71. 25201189 10.1007/978-94-017-9211-0_4

[46] E. Zlotorynski , Nat. Rev. Mol. Cell Biol. 2023, 24, 4. 10.1038/s41580-023-00596-w 36914762

[47] Y. Nishida , V. Impedovo , E. Ayoub , bioRxiv 2025, 650490.

[48] S. Nair , N. Bora‐Singhal , D. Perumal , S. Chellappan , Mol Cancer 2014, 13, 173. 25028095 10.1186/1476-4598-13-173 PMC4121302

[49] W. Sun , L. Zhang , R. Yan , Y. Yang , X. Meng , Onco. Targets Ther. 2019, 12, 3945. 31190891 10.2147/OTT.S196865 PMC6535439

[50] Y. Cheng , S. Wang , X. Mu , Transl. Cancer Res. TCR 2021, 10, 5159. 35116366 10.21037/tcr-21-1468 PMC8798158

[51] X. Chen , C. Feng , J. Zha , Z. Shen , W. Ji , Acta Biochim. Pol. 2023, 70, 835. 38099479 10.18388/abp.2020_6660

[52] Z. Li , X. Xie , X. Fan , X. Li , Neurochem. Res. 2020, 45, 1690. 32333234 10.1007/s11064-020-03029-8

[53] W. Wu , L. Guo , Z. Liang , Y. Liu , Z. Yao , J. Cancer 2020, 11, 2201. 32127947 10.7150/jca.40319 PMC7052928

[54] Q.‐G. Tian , R.‐C. Tian , Y. Liu , A.‐Y. Niu , J. Zhang , W.‐F. Gao , Eur. Rev. Med. Pharmacol. Sci. 2018, 22, 4138. 30024602 10.26355/eurrev_201807_15406

[55] C. Zhang , Y. Zou , D.‐Q. Dai , Biomed. Pharmacother. 2019, 119, 109417. 31539861 10.1016/j.biopha.2019.109417

[56] E. Gottwein , D. L. Corcoran , N. Mukherjee , R. L. Skalsky , M. Hafner , J. D. Nusbaum , P. Shamulailatpam , C. L. Love , S. S. Dave , T. Tuschl , U. Ohler , B. R. Cullen , Cell Host Microbe. 2011, 10, 515. 22100165 10.1016/j.chom.2011.09.012 PMC3222872

[57] starBase or ENCORI: Decoding the Encyclopedia of RNA Interactomes, https://rnasysu.com/encori/ (accessed: November 2025).

[58] cBioPortal for Cancer Genomics, https://www.cbioportal.org/ (accessed: November 2025).

[59] J. Yang , Y. Li , A. Aguilar , Z. Liu , C.‐Y. Yang , S. Wang , J. Med. Chem. 2019, 62, 9471. 31560543 10.1021/acs.jmedchem.9b00846 PMC7354697

[60] T. Bilbrough , E. Piemontese , O. Seitz , Chem. Soc. Rev. 2022, 51, 5691. 35726784 10.1039/d1cs00991e

[61] I. Diallo , M. Seve , V. Cunin , F. Minassian , J.‐F. Poisson , S. Michelland , S. Bourgoin‐Voillard , Expert Rev. Proteomics 2019, 16, 139. 30580641 10.1080/14789450.2019.1559061

[62] Y. Kikuchi , H. Shimatake , A. Kikuchi , EMBO J. 1988, 7, 1175. 2841115 10.1002/j.1460-2075.1988.tb02928.x PMC454453

[63] S. Kervestin , A. Jacobson , Nat. Rev. Mol. Cell Biol. 2012, 13, 700. 23072888 10.1038/nrm3454 PMC3970730

[64] K. M. Hannan , E. Sanij , L. I. Rothblum , R. D. Hannan , R. B. Pearson , Bioch. Biophys. Acta 2013, 1829, 342. 10.1016/j.bbagrm.2012.10.014 PMC3594452 23153826

[65] A. Yamashita , Genes Cells 2013, 18, 161. 23356578 10.1111/gtc.12033

[66] E. Z. Alkalaeva , A. V. Pisarev , L. Y. Frolova , L. L. Kisselev , T. V. Pestova , Cell 2006, 125, 1125. 16777602 10.1016/j.cell.2006.04.035

[67] V. P. Pisareva , A. V. Pisarev , C. U. T. Hellen , M. V. Rodnina , T. V. Pestova , J. Biol. Chem. 2006, 281, 40224. 17062564 10.1074/jbc.M607461200

[68] V. A. Mitkevich , A. V. Kononenko , I. Y. Petrushanko , D. V. Yanvarev , A. A. Makarov , L. L. Kisselev , Nucleic Acids Res. 2006, 34, 3947. 16914449 10.1093/nar/gkl549 PMC1557817

[69] M. R. Lawson , L. N. Lessen , J. Wang , A. Prabhakar , N. C. Corsepius , R. Green , J. D. Puglisi , Science. 2021, 373, 876. 34413231 10.1126/science.abi7801 PMC9017434

[70] C. U. T. Hellen , Cold Spring Harb. Perspect. Biol. 2018, 10, a032656. 29735640 10.1101/cshperspect.a032656 PMC6169810

[71] R. Hegde , S. M. Srinivasula , P. Datta , M. Madesh , R. Wassell , Z. Zhang , N. Cheong , J. Nejmeh , T. Fernandes‐Alnemri , S.‐I. Hoshino , E. S. Alnemri , J. Biol. Chem. 2003, 278, 38699. 12865429 10.1074/jbc.M303179200

[72] A. M. Verhagen , T. K. Kratina , C. J. Hawkins , J. Silke , P. G. Ekert , D. L. Vaux , Cell Death Differ. 2007, 14, 348. 16794601 10.1038/sj.cdd.4402001

[73] Y. Hashimoto , H. Inagaki , S. Hoshino , FEBS Lett. 2015, 589, 2241. 26172506 10.1016/j.febslet.2015.06.041

[74] R. Xiao , Y. Gao , Q. Shen , C. Li , W. Chang , B. Chai , Cell Biol. Int. 2013, 37, 359. 23377885 10.1002/cbin.10043

[75] Y. Hashimoto , N. Kumagai , N. Hosoda , S. Hoshino , Biochem. Biophys. Res. Commun. 2014, 445, 639. 24569073 10.1016/j.bbrc.2014.02.063

[76] J. A. Lee , J. E. Park , D. H. Lee , S. G. Park , P. K. Myung , B. C. Park , S. Cho , Oncogene 2008, 27, 1297. 17700517 10.1038/sj.onc.1210740

[77] T. Kitamura , Y. Fukuyo , M. Inoue , N. T. Horikoshi , M. Shindoh , B. E. Rogers , A. Usheva , N. Horikoshi , Cancer Res. 2009, 69, 7681. 19789335 10.1158/0008-5472.CAN-09-2133 PMC2855193

[78] S.‐S. Lai , D.‐D. Zhao , P. Cao , K. Lu , O.‐Y. Luo , W.‐B. Chen , J. Liu , E.‐Z. Jiang , Z.‐H. Yu , G. Lee , J. Li , D.‐C. Yu , X.‐J. Xu , M.‐S. Zhu , X. Gao , C.‐J. Li , B. Xue , J. Hepatol. 2016, 64, 352. 26456844 10.1016/j.jhep.2015.09.025

[79] R. G. H. Lindeboom , M. Vermeulen , B. Lehner , F. Supek , Nat. Genet. 2019, 51, 1645. 31659324 10.1038/s41588-019-0517-5 PMC6858879

[80] G. Gavory , M. Ghandi , A.‐C. d'Alessandro , D. Bonenfant , A. Chicas , F. Delobel , B. Demarco , A. Flohr , C. King , A.‐L. Laine , V. Massafra , R. Narayan , A. Osmont , G. Ottaviani , D. Peck , S. Pessa , N. Rubin , T. Ryckmans , M. Schillo , A. Singh , S. Tortoioli , D. Vigil , V. Zarayskiy , J. Castle , F. Janku , O. Wallace , S. Buonamici , B. Fasching , Cancer Res. 2022, 82, 3929.

[81] C. Chauvin , S. Salhi , O. Jean‐Jean , Mol. Cell. Biol. 2007, 27, 5619. 17562865 10.1128/MCB.00035-07 PMC1952125

[82] R. S. Sellar , A. S. Sperling , M. Slabicki , J. A. Gasser , M. E. McConkey , K. A. Donovan , N. Mageed , D. N. Adams , C. Zou , P. G. Miller , R. K. Dutta , S. Boettcher , A. E. Lin , B. Sandoval , V. A. Quevedo Barrios , V. Kovalcik , J. Koeppel , E. K. Henderson , E. C. Fink , L. Yang , A. Chan , S. P. Pokharel , E. J. Bergstrom , R. Burt , N. D. Udeshi , S. A. Carr , E. S. Fischer , C.‐W. Chen , B. L. Ebert , J. Clin. Invest. 2022, 132, 153514. 10.1172/JCI153514 PMC9374383 35763353

[83] M. Li , J. Wang , L. Yang , P. Gao , Q. Tian , D. Liu , PLoS One 2014, 9, 86371. 10.1371/journal.pone.0086371 PMC3900531 24466059

[84] T. Ishii , T. Ueyama , M. Shigyo , M. Kohta , T. Kondoh , T. Kuboyama , T. Uebi , T. Hamada , D. H. Gutmann , A. Aiba , E. Kohmura , C. Tohda , N. Saito , J. Biol. Chem. 2017, 292, 1240. 27941025 10.1074/jbc.M116.748871 PMC5270470

[85] T. Ueyama , Cells 2019, 8, 92. 30696065 10.3390/cells8020092 PMC6406560

[86] A. Lopez‐Girona , G. Lu , E. Rychak , D. Mendy , C.‐C. Lu , I. Rappley , C. Fontanillo , B. E. Cathers , T. O. Daniel , J. Hansen , Blood 2019, 134, 2703.

[87] G. Gavory , M. Ghandi , A.‐C. d'Alessandro , D. Bonenfant , M. Cabanski , L. Cantagallo , A. Chicas , Q. Chen , A. Diesslin , C. King , V. Massafra , R. Narayan , A. Osmont , D. Peck , C. P. Ortiz , M. Schillo , A. Singh , R. Tiedt , S. Tortoioli , S. Buonamici , F. Janku , O. Wallace , B. Fasching , Cancer Res. 2023, 83, 3449.

[88] M. Mijit , M. Boner , R. A. Cordova , S. Gampala , E. Kpenu , A. J. Klunk , C. Zhang , M. R. Kelley , K. A. Staschke , M. L. Fishel , Front. Med. 2023, 10, 1146115. 10.3389/fmed.2023.1146115 PMC10174294 37181357

[89] Y. Tatara , S. Kasai , D. Kokubu , T. Tsujita , J. Mimura , K. Itoh , Int. J. Mol. Sci. 2024, 25, 2998. 38474243 10.3390/ijms25052998 PMC10931611

[90] X. Tian , W. S. El‐Deiry , Oncotarget 2024, 15, 614. 39288289 10.18632/oncotarget.28637 PMC11407758

[91] K. Pakos‐Zebrucka , I. Koryga , K. Mnich , M. Ljujic , A. Samali , A. M. Gorman , EMBO Rep. 2016, 17, 1374. 27629041 10.15252/embr.201642195 PMC5048378

[92] M. J. Nutt , S. G. Stewart , Drug Discovery Today 2024, 29, 104010. 38704021 10.1016/j.drudis.2024.104010

[93] B.‐B. Hao , X.‐J. Li , X.‐L. Jia , Y.‐X. Wang , L.‐H. Zhai , D.‐Z. Li , J. Liu , D. Zhang , Y.‐L. Chen , Y.‐H. Xu , S.‐K. Lee , G.‐F. Xu , X.‐H. Chen , Y.‐J. Dang , B. Liu , M.‐J. Tan , Acta Pharmacol. Sin. 2020, 41, 1246. 32210356 10.1038/s41401-020-0367-9 PMC7608331

[94] S. Lier , A. Sellmer , F. Orben , S. Heinzlmeir , L. Krauß , C. Schneeweis , Z. Hassan , C. Schneider , A. Patricia Gloria Schäfer , H. Pongratz , T. Engleitner , R. Öllinger , A. Kuisl , F. Bassermann , C. Schlag , B. Kong , S. Dove , B. Kuster , R. Rad , M. Reichert , M. Wirth , D. Saur , S. Mahboobi , G. Schneider , Bioorg. Chem. 2022, 119, 105505. 34838332 10.1016/j.bioorg.2021.105505

[95] B. Wang , J. Liu , I. Tandon , S. Wu , P. Teng , J. Liao , W. Tang , Eur. J. Med. Chem. 2021, 219, 113425. 33862513 10.1016/j.ejmech.2021.113425 PMC8165035

[96] J. Zou , R. J. Jones , H. Wang , I. Kuiatse , F. Shirazi , E. E. Manasanch , H. C. Lee , R. Sullivan , L. Fung , N. Richard , P. Erdman , E. Torres , D. Hecht , I. Lam , B. McElwee , A. H. Chourasia , K. W. H. Chan , F. Mercurio , D. I. Stirling , R. Z. Orlowski , J. Mol. Med. 2020, 98, 1161. 32632752 10.1007/s00109-020-01943-6 PMC10838157

[97] L. Ma , Y. Tong , Z. Yang , Q. Zhou , H. Yan , R. Xu , D. Chen , Cancer Res. 2024, 84, 3297.

[98] Captor Therapeutics, https://captortherapeutics.com/ (accessed: November 2025).

[99] J. Zhai , C. Li , S. Wang , B. Sun , Y. Cui , Q. Gao , F. Sang , ChemistrySelect 2022, 7, 202203463.

[100] Y. Yang , Q. Yao , D. Song , K. Tang , Z. Tang , M. Hu , Y. Luo , Y. Xie , Eur. J. Med. Chem. 2025, 296, 117893. 40578254 10.1016/j.ejmech.2025.117893

[101] V. Oleinikovas , P. Gainza , T. Ryckmans , B. Fasching , N. H. Thomä , Annu. Rev. Pharmacol. Toxicol. 2024, 64, 291. 37585660 10.1146/annurev-pharmtox-022123-104147

[102] P. Glaza , R. Pluta , K. E. Odrzywół , M. Klejnot , M. Wieczorek , S. Cottens , D. Coppen , P. Dobrzański , T. Drmota , J. Lis‐Grześniak , A. Śnieżewska , J. Majkut , M. Mianowska , P. Rozborska , M. Jarmuszkiewicz , K. Kaczanowska , A. Adamska , T. Takagi , A. Sawicka , A. Serwotka‐Suszczak , O. Makowska , D. Gajewska , K. Jurczak , K. Leszkowicz , M. Mankiewicz , K. Przytulski , J. Wiśniewski , A. Szlachcic , M. J. Walczak , Commun. Chem. 2025, 8, 247. 40813917 10.1038/s42004-025-01641-9 PMC12354682

[103] J. Fan , H. Wang , S. Couto , T.‐W. S. Yao , G. L. Uy , A. M. Zeidan , M. D. Minden , P. Montesinos , D. J. DeAngelo , J. K. Altman , J. Koprivnikar , P. Vyas , Y. Fløisand , M. Belén Vidriales , B. T. Gjertsen , T. J. Buchholz , M. Pourdehnad , D. W. Pierce , Blood 2019, 134, 2547.

[104] J. D. Hansen , M. Correa , M. Alexander , M. Nagy , D. Huang , J. Sapienza , G. Lu , L. A. LeBrun , B. E. Cathers , W. Zhang , Y. Tang , M. Ammirante , R. K. Narla , J. R. Piccotti , M. Pourdehnad , A. Lopez‐Girona , J. Med. Chem. 2021, 64, 1835. 33591756 10.1021/acs.jmedchem.0c01489

[105] G. L. Uy , M. D. Minden , P. Montesinos , D. J. DeAngelo , J. K. Altman , J. Koprivnikar , P. Vyas , Y. Fløisand , M. Belén Vidriales , B. T. Gjertsen , J. Esteve , T. J. Buchholz , S. Couto , J. Fan , B. Hanna , L. Li , D. W. Pierce , K. Hege , M. Pourdehnad , A. M. Zeidan , Blood 2019, 134, 232.

[106] Y. Zhang , W. Liu , C. Tong , X. Wang , X. Chang , F. Qu , Z. Zhang , Z. Fan , M. Zhao , C. Tang , B. Song , M. Ding , Z. Qiu , J. Wang , J. Bian , Z. Li , H. Wu , X. Xu , J. Med. Chem. 2025, 68, 2608. 39854008 10.1021/acs.jmedchem.4c01787

[107] C.‐C. Chan , C.‐S. Li , L. Hu , W. Li , Z. Zhu , S. Chen , Cancer Res. 2025, 85, 393.

[108] R. Tiedt , M. Schillo , A. Osmont , D. Bonenfant , R. Narayan , O. Wallace , M. Warmuth , Cancer Res. 2024, 84, 3294.

[109] M. Rosa Therapeutics, https://www.monterosatx.com/ (accessed: November 2025).

[110] N. P. Taylor , Monte Rosa's *Broad Molecular Glu*e degrader *Plan Comes Unst*uck, FIERCE Biotech, New York US 2025.

[111] Center For Drug Evaluation, NMPA, https://www.cde.org.cn/ (accessed: November 2025).

[112] A. D. Huber , Y. Li , W. Lin , A. N. Galbraith , A. Mishra , S. N. Porter , J. Wu , R. R. Florke Gee , W. Zhuang , S. M. Pruett‐Miller , J. Peng , T. Chen , ACS Med. Chem. Lett. 2022, 13, 1311. 35978691 10.1021/acsmedchemlett.2c00223 PMC9377019

[113] H. Shen , H. Xu , W. Jin , T. Wu , J. Hu , C. Zhang , Z. Zhong , J. Li , R. Mao , S. Zhang , X. Zhang , X. Wu , J. B. Smaill , J. Xu , Y. Zhang , Y. Xu , J. Med. Chem. 2025, 68, 1553. 39746330 10.1021/acs.jmedchem.4c02205

[114] CYRS1542 / Cyrus Therap, https://delta.larvol.com/Products/?ProductId=008fd8bc‐93d3‐4eca‐85d4‐ab7388ed24e2&Index=0 (accessed: November 2025).

[115] J. Park , M. S. Joo , J. Lee , E.‐J. Kim , D. H. Ki , H. Choi , W. Han , K. W. Kang , Cancer Res. 2025, 85, 386.

[116] Y. Wei , X. Xu , M. Jiang , Y. Wang , Y. Zhou , Z. Wang , Z. Zhang , F. Zhou , K. Ding , Eur. J. Med. Chem. 2023, 258, 115580. 37418973 10.1016/j.ejmech.2023.115580

[117] J. M. Sasso , R. Tenchov , D. Wang , L. S. Johnson , X. Wang , Q. A. Zhou , Biochemistry 2023, 62, 601. 35856839 10.1021/acs.biochem.2c00245 PMC9910052

[118] M. Ishoey , S. Chorn , N. Singh , M. G. Jaeger , M. Brand , J. Paulk , S. Bauer , M. A. Erb , K. Parapatics , A. C. Müller , K. L. Bennett , G. F. Ecker , J. E. Bradner , G. E. Winter , ACS Chem. Biol. 2018, 13, 553. 29356495 10.1021/acschembio.7b00969

[119] M. S. Joo , J. Lee , E.‐J. Kim , D. H. Ki , H. Choi , J. Nam , J. Park , K. W. Kang , W. Han , Cancer Res. 2023, 83, 1673.

[120] S. Zhang , S. Nie , G. Ma , M. Shen , L. Kong , Z. Zuo , Y. Li , Eur. J. Med. Chem. 2024, 273, 116524. 38795517 10.1016/j.ejmech.2024.116524

[121] Y. Tang , H. Wang , J. Zhang , C. Yang , F. Xu , Y. Song , T. Li , Q. Zhang , Sci. Rep. 2025, 15, 2477. 39833282 10.1038/s41598-025-86185-7 PMC11747321

[122] J. Zhao , S. Agarwal , H. Ahmad , K. Amin , J. P. Bewersdorf , A. Zeidan , Blood Rev. 2022, 52, 100905. 34774343 10.1016/j.blre.2021.100905 PMC9846716

[123] D. W. Pierce , T.‐W. S. Yao , E. Pace , H. Wang , P. Flandin‐Blety , A. Benitez , C. Guarinos , M. Hoffmann , S. Carrancio , J. Fan , M. Pourdehnad , Blood 2021, 138, 3330.

[124] E. M. Stein , Blood 2023, 141, 124.36633887

[125] A. W. Roberts , Hematology 2020, 2020, 1. 33275682 10.1182/hematology.2020000154 PMC7727569

[126] E. J. Vick , A. Hassan , K. Choi , J. Bennett , T. Muto , C. A. Clough , A. E. Culver‐Cochran , K. Hueneman , L. C. Bolanos , M. Wunderlich , X. Zhang , C. McKnight , M. Ceribelli , D. Holland , C. Klumpp‐Thomas , K. D. Greis , C. J. Thomas , D. T. Starczynowski , Leukemia 2025, 39, 2163. 40670672 10.1038/s41375-025-02695-3 PMC12380595

[127] Y. Nishida , D. A. Scruggs , E. Ayoub , T. Patsilevas , V. R. Ruvolo , P. Y. Mak , B. Z. Carter , S. Boettcher , A. Maiti , Q. Zhou , Z. Yang , H. Yan , L. Ma , M. Andreeff , Clin. Lymph., Myel. Leuk. 2022, 22, S218.

[128] L. Ma , Y. Tong , Z. Yang , Q. Zhou , H. Yan , R. Xu , J. Chen , J. Pan , H. Wang , J. Li , D. Chen , X. Cai , J. Qu , Y. Wang , J. Qin , Y. Nishida , M. Andreeff , Q. Guo , Y. Nishida , M. Andreeff , Cancer Res. 2022, 82, 5479.

[129] M. Tang , J. Crown , M. J. Duffy , Invest New Drugs 2025, 43, 167. 39875774 10.1007/s10637-024-01504-5 PMC11868176

[130] Z. Zhang , M. Chen , C. Cai , L. Teng , S. Liu , Y. Chang , X. Jin , X. Zhang , D. Liu , Cancer Res. 2025, 85, 383.

[131] G. Nishiguchi , F. Keramatnia , J. Min , Y. Chang , B. Jonchere , S. Das , M. Actis , J. Price , D. Chepyala , B. Young , K. McGowan , P. J. Slavish , A. Mayasundari , J. A. Jarusiewicz , L. Yang , Y. Li , X. Fu , S. H. Garrett , J. B. Papizan , K. Kodali , J. Peng , S. M. Pruett Miller , M. F. Roussel , C. Mullighan , M. Fischer , Z. Rankovic , J. Med. Chem. 2021, 64, 7296. 34042448 10.1021/acs.jmedchem.0c01313 PMC8201443

[132] Y. Chang , F. Keramatnia , P. S. Ghate , G. Nishiguchi , Q. Gao , I. Iacobucci , L. Yang , D. Chepyala , A. Mishra , A. A. High , H. Goto , K. Akahane , J. Peng , J. J. Yang , M. Fischer , Z. Rankovic , C. G. Mullighan , Blood 2023, 142, 629. 37172201 10.1182/blood.2022017813 PMC10447621

[133] Y. Feng , X. Hu , X. Wang , Biomark. Res. 2024, 12, 85. 39169396 10.1186/s40364-024-00638-1 PMC11340087

[134] C. Zhang , B. Jiang , X. Liang , Y. Chen , Z. Li , M. Zhao , D. Lin , Anticancer Agents Med. Chem. 2025. 10.2174/0118715206407523250902055051 41029930

[135] A. H. Chourasia , H. Majeski , A. Pasis , P. Erdman , A. Oke , D. Hecht , D. Lonergan , F. Mercurio , K. Chan , D. L. Thai , L. Fung , JCO 2022, 40, 7025.

[136] Z. Wang , S. Shaabani , X. Gao , Y. L. D. Ng , V. Sapozhnikova , P. Mertins , J. Krönke , A. Dömling , Nat. Commun. 2023, 14, 8437. 38114468 10.1038/s41467-023-43614-3 PMC10730884

[137] F. Xiong , L. Kong , L. Chen , M. Xue , F. Cao , S. Zhang , H. Li , H. Yan , Y. Li , Z. Zuo , Eur. J. Med. Chem. 2022, 236, 114355. 35413617 10.1016/j.ejmech.2022.114355

[138] A. D. Takwale , E. Y. Kim , Y. Jang , D. H. Lee , S. Kim , Y. Choi , J. H. Kim , D. Y. Lee , Y. Kim , S. M. Lee , H. K. Lee , H. J. Nam , J.‐Y. Lee , J. H. Cho , J. H. Moon , G. S. Lee , J.‐H. Kim , P. Kim , C. H. Park , J. Y. Hwang , Bioorg. Chem. 2022, 127, 105923. 35717803 10.1016/j.bioorg.2022.105923

[139] FenDi Pharmaceutical, http://www.prodedrug.com/pipline/ (accessed: November 2025).

[140] J. Zuber , J. Shi , E. Wang , A. R. Rappaport , H. Herrmann , E. A. Sison , D. Magoon , J. Qi , K. Blatt , M. Wunderlich , M. J. Taylor , C. Johns , A. Chicas , J. C. Mulloy , S. C. Kogan , P. Brown , P. Valent , J. E. Bradner , S. W. Lowe , C. R. Vakoc , Nature 2011, 478, 524. 21814200 10.1038/nature10334 PMC3328300

[141] Y. Xu , H. Yang , Y. Li , Y. Qi , F. Zhao , Y. Hong , B. Cheng , Z. Lu , J. Zhang , C. Guo , J. Fu , Q. Lin , C. Chen , N. Shi , J. Cai , K. Li , S. Wang , R. Gao , D. Dai , Eur. J. Med. Chem. 2025, 288, 117381. 39965406 10.1016/j.ejmech.2025.117381

[142] L. J. Alcock , Y. Chang , J. A. Jarusiewicz , M. Actis , S. Nithianantham , A. Mayasundari , J. Min , D. Maxwell , J. Hunt , B. Smart , J. J. Yang , G. Nishiguchi , M. Fischer , C. G. Mullighan , Z. Rankovic , ACS Med. Chem. Lett. 2022, 13, 475. 35300081 10.1021/acsmedchemlett.1c00650 PMC8919382

[143] Y. Chang , J. Min , J. A. Jarusiewicz , M. Actis , S. Yu‐Chen Bradford , A. Mayasundari , L. Yang , D. Chepyala , L. J. Alcock , K. G. Roberts , S. Nithianantham , D. Maxwell , L. Rowland , R. Larsen , A. Seth , H. Goto , T. Imamura , K. Akahane , B. S. Hansen , S. M. Pruett‐Miller , E. M. Paietta , M. R. Litzow , C. Qu , J. J. Yang , M. Fischer , Z. Rankovic , C. G. Mullighan , Blood 2021, 138, 2313. 34110416 10.1182/blood.2020006846 PMC8662068

[144] I. Tandon , P. N. Esguerra , C. Li , H. Sun , K. Yang , Z. Zhang , J. Peng , W. Tang , Eur. J. Med. Chem. 2025, 295, 117793. 40440791 10.1016/j.ejmech.2025.117793 PMC12146042

[145] Z. Yang , Y. Sun , Z. Ni , C. Yang , Y. Tong , Y. Liu , H. Li , Y. Rao , Cell Res. 2021, 31, 1315. 34417569 10.1038/s41422-021-00533-6 PMC8648895

[146] R. E. Davis , V. N. Ngo , G. Lenz , P. Tolar , R. M. Young , P. B. Romesser , H. Kohlhammer , L. Lamy , H. Zhao , Y. Yang , W. Xu , A. L. Shaffer , G. Wright , W. Xiao , J. Powell , J.‐K. Jiang , C. J. Thomas , A. Rosenwald , G. Ott , H. K. Muller‐Hermelink , R. D. Gascoyne , J. M. Connors , N. A. Johnson , L. M. Rimsza , E. Campo , E. S. Jaffe , W. H. Wilson , J. Delabie , E. B. Smeland , R. I. Fisher , et al., Nature 2010, 463, 88. 20054396 10.1038/nature08638 PMC2845535

[147] B. Zhang , S. Gao , T. Wu , J. Med. Chem. 2025.

[148] C. Maracci , S. Motta , A. Romagnoli , M. Costantino , P. Perego , D. Di Marino , Curr. Med. Chem. 2022, 29, 3501. 35209811 10.2174/0929867329666220224112042

[149] Y. Liu , X. Sun , Q. Liu , C. Han , Y. Rao , J. Am. Chem. Soc. 2025, 147, 3110. 39622049 10.1021/jacs.4c11930

[150] P. S. Dragovich , Chem. Soc. Rev. 2022, 51, 3886. 35506708 10.1039/d2cs00141a

[151] J. Venkatesan , D. Murugan , K. Lakshminarayanan , A. R. Smith , H. Vasanthakumari Thirumalaiswamy , H. Kandhasamy , B. Zender , G. Zheng , L. Rangasamy , Pharmacol. Ther. 2024, 263, 108725. 39322067 10.1016/j.pharmthera.2024.108725

[152] S. A. Hurvitz , E. P. Hamilton , A. I. Spira , P. R. Pohlmann , JCO 2023, 41, TPS1114.

[153] J. Palacino , C. Bai , Y. Yi , A. Skaletskaya , K. Takrouri , W. Wong , M.‐S. Kim , D.‐K. Choi , D.‐Y. Kim , Y. Yang , J. Kook , P. Lee , H. Jeong , S.‐M. Jee , J. Park , K.‐H. Chang , N. Fishkin , P. U. Park , Cancer Res. 2022, 82, 3933.

[154] J. Palacino , P. Lee , H. Jeong , Y. Kim , Y. Song , U. Permpoon , W. Wong , C. Bai , N. Fishkin , K. Takrouri , E. Yu , Y. Yi , A. Skaletskaya , K.‐H. Chang , M.‐S. Kim , D.‐Y. Kim , D.‐K. Choi , P. U. Park , Blood 2022, 140, 3061.

[155] Orum Therapeutics Provides Program Update and Announces Drug Candidate Nomination, https://www.orumrx.com/news/orum‐therapeutics‐provides‐program‐update‐and‐announces‐drug‐candidate‐nomination (accessed: November 2025).

[156] H. K. Lee , S. Kim , D. Kim , S. Choi , J. Lee , S. Lee , E. Choi , S. W. Park , K. Chun , K. Lee , Cancer Res. 2025, 85, 382.

[157] E. C. Fink , M. McConkey , D. N. Adams , S. D. Haldar , J. A. Kennedy , A. A. Guirguis , N. D. Udeshi , D. R. Mani , M. Chen , B. Liddicoat , T. Svinkina , A. T. Nguyen , S. A. Carr , B. L. Ebert , Blood 2018, 132, 1535. 30064974 10.1182/blood-2018-05-852798 PMC6172563

[158] biocytogen, https://biocytogen.com.cn/ (accessed: November 2025).

[159] M. J. Mattes , Cancer 2002, 94, 1215. 11877748 10.1002/cncr.10288

[160] M. Alas , A. Saghaeidehkordi , K. Kaur , J. Med. Chem. 2021, 64, 216. 33382619 10.1021/acs.jmedchem.0c01530 PMC8610607

[161] J. Tao , Y. Gu , W. Zhou , Y. Wang , Eur. J. Med. Chem. 2025, 281, 116995. 39481229 10.1016/j.ejmech.2024.116995

[162] DepMap: The Cancer Dependency Map Project at Broad Institute, https://depmap.org/portal/ (accessed: November 2025).

[163] J. Park , M. S. Joo , M. J. Kim , S. Oh , P. T. Tran , M. Kwon , Y. J. Choi , J. Lee , E.‐J. Kim , D. H. Ki , H. Choi , W. Han , K. W. Kang , Exp. Hematol. Oncol. 2025, 14, 89. 40551250 10.1186/s40164-025-00674-z PMC12186427

[164] R. Ravindran , A. K. G. Velikkakath , N. D. Narendradev , A. Chandrasekharan , T. R. Santhoshkumar , S. M. Srinivasula , Autophagy 2022, 18, 2851. 35373701 10.1080/15548627.2022.2052460 PMC9673925

[165] Q. Yang , J. Zhao , D. Chen , Y. Wang , Mol. Biomed. 2021, 2, 23. 35006464 10.1186/s43556-021-00043-2 PMC8607428

[166] J. Qi , H. Kim , M. Scortegagna , Z. A. Ronai , Cell Biochem. Biophys. 2013, 67, 15. 23700162 10.1007/s12013-013-9636-2 PMC3758783

[167] C. Wang , Y. Zhang , J. Wang , D. Xing , Eur. J. Med. Chem. 2022, 227, 113906. 34656901 10.1016/j.ejmech.2021.113906

[168] R. M. Ewing , P. Chu , F. Elisma , H. Li , P. Taylor , S. Climie , L. McBroom‐Cerajewski , M. D. Robinson , L. O'Connor , M. Li , R. Taylor , M. Dharsee , Y. Ho , A. Heilbut , L. Moore , S. Zhang , O. Ornatsky , Y. V. Bukhman , M. Ethier , Y. Sheng , J. Vasilescu , M. Abu‐Farha , J.‐P. Lambert , H. S. Duewel , I. I. Stewart , B. Kuehl , K. Hogue , K. Colwill , K. Gladwish , B. Muskat , et al., Mol. Syst. Biol. 2007, 3, 89. 17353931 10.1038/msb4100134 PMC1847948

[169] M. J. R. Yeo , O. Zhang , X. Xie , E. Nam , N. C. Payne , P. M. Gosavi , H. S. Kwok , I. Iram , C. Lee , J. Li , N. J. Chen , K. Nguyen , H. Jiang , Z. A. Wang , K. Lee , H. Mao , S. A. Harry , I. A. Barakat , M. Takahashi , A. L. Waterbury , M. Barone , A. Mattevi , S. A. Carr , N. D. Udeshi , L. Bar‐Peled , P. A. Cole , R. Mazitschek , B. B. Liau , N. Zheng , Nature 2025, 639, 232. 39939761 10.1038/s41586-024-08532-4 PMC11882444

[170] The Human Protein Atlas, https://www.proteinatlas.org/ (accessed: November 2025).

[171] A. S. Pathania , P. Prathipati , S. P. Murakonda , A. B. Murakonda , A. Srivastava , Avadhesh , S. N. Byrareddy , D. W. Coulter , S. C. Gupta , K. B. Challagundla , Semin. Cancer Biol. 2022, 86, 247. 35787940 10.1016/j.semcancer.2022.06.013

[172] X. Chang , F. Qu , C. Li , J. Zhang , Y. Zhang , Y. Xie , Z. Fan , J. Bian , J. Wang , Z. Li , X. Xu , Med. Res. Rev. 2024, 44, 1727. 38314926 10.1002/med.22024

[173] S. A. Ochsner , D. Abraham , K. Martin , Sci. Data 2019, 6, 252. 31672983 10.1038/s41597-019-0193-4 PMC6823428

[174] J. Palacino , P. Lee , H. Jeong , Y. Kim , Y. Song , U. Permpoon , W. Wong , C. Bai , N. Fishkin , K. Takrouri , E. Yu , Y. Yi , A. Skaletskaya , K.‐H. Chang , M.‐S. Kim , D.‐Y. Kim , D.‐K. Choi , P. U. Park , Blood 2022, 140, 3061.

[175] J. He , J. Guo , S. Liu , H. Li , Y. Ma , S. Ma , Z. Hu , W. Zhao , M. Tan , W. Liu , B. Liu , Cell. Signal. 2025, 130, 111665. 39986359 10.1016/j.cellsig.2025.111665

[176] L. Ma , Y. Tong , Z. Yang , Cancer Res. 2024, 84.37874330

[177] G. Gavory , B. Fasching , D. Bonenfant , A. Sadok , A. Singh , M. Schillo , V. Massafra , A.‐C. d'Alessandro , J. Castle , M. Ghandi , A. Chicas , F. Delobel , A. Flohr , G. Ottaviani , T. Ryckmans , A.‐L. Laine , O. Eidam , H. Wang , I. Bernett , L. Chan , C. Gorrini , T. Roumiliotis , J. Choudhary , Y.‐V. LeBihan , M. Cabry , M. Stubbs , R. Burke , R. Van Montfort , J. Caldwell , R. Chopra , et al., Mol. Cancer Ther. 2021, 20, LBA004.

