# Supplementary material for: Cancer Biology of GSPT1: Mechanisms and Targeted Therapy Opportunities of Molecular Glue Degraders
Source: Adv Sci (Weinh). 2025 Nov 5;12(47):e11789. doi: 10.1002/advs.202511789 (PMC12713026; doi:10.1002/advs.202511789)
Supplement: Supplementary file 1 — Supporting Information [file ADVS-12-e11789-s001.docx]

**Cancer Biology of GSPT1: Mechanisms and Targeted Therapy Opportunities of Molecular Glue Degraders**

Qiqi Lin^1, #^, Wenjing Liu^1, #^, Wenjia Lu^2^, Monong Zhao^1^, Lin Cao^1^, Zhiyu Li^3, *^, Jubo Wang^3, *^, Xi Xu^3, *^, Hongxi Wu^1, *^

1. State Key Laboratory of Natural Medicines, Department of Pharmacology, School of Pharmacy, China Pharmaceutical University, Nanjing 211198, China.
2. School of International Pharmaceutical Business, China Pharmaceutical University, Nanjing 211198, China.
3. State Key Laboratory of Natural Medicines, Jiangsu Key Laboratory of Drug Design and Optimization, Department of Medicinal Chemistry, School of Pharmacy, China Pharmaceutical University, Nanjing 211198, China.

**Correspondence:**

whx@cpu.edu.cn; xuxi@cpu.edu.cn; 1620194588@cpu.edu.cn; zhiyuli@cpu.edu.cn

^#^The authors contributed equally.

**Authors information:**

**Qiqi Lin**, Department of Pharmacology, School of Pharmacy, China Pharmaceutical University,

Nanjing 211100, P.R. China.

Email: lqq0772002@163.com

**Wenjing Liu**, Department of Pharmacology, School of Pharmacy, China Pharmaceutical University,

Nanjing 211100, P.R. China.

Email: 17860505453@163.com

**Wenjia Lu**, School of International Pharmaceutical Business, China Pharmaceutical University,

Nanjing 211100, P.R. China.

Email: 15848909826@163.com

**Monong Zhao**, Department of Pharmacology, School of Pharmacy, China Pharmaceutical University,

Nanjing 211100, P.R. China.

Email: zhaomonong1536@163.com

**Lin Cao**, Department of Pharmacology, School of Pharmacy, China Pharmaceutical University,

Nanjing 211100, P.R. China.

Email: [caolin10112024@163.com](mailto:caolin10112024@163.com)

**Zhiyu Li**, Department of Medicinal Chemistry, China Pharmaceutical University,

Nanjing 211100, P. R. China

Email: [zhiyuli@cpu.edu.cn](mailto:zhiyuli@cpu.edu.cn)

**Jubo Wang**, Department of Medicinal Chemistry, China Pharmaceutical University,

Nanjing 211100, P. R. China

E-mail: [1620194588@cpu.edu.cn](mailto:1620194588@cpu.edu.cn)

**Xi Xu**, Department of Medicinal Chemistry, China Pharmaceutical University,

Nanjing 211100, P. R. China

E-mail: xuxi@cpu.edu.cn

**Hongxi Wu**, Department of Pharmacology, School of Pharmacy, China Pharmaceutical University,

Nanjing 211100, P.R. China.

Email: [whx@cpu.edu.cn](mailto:whx@cpu.edu.cn)

**Table S1 GSPT1/ERF3a gene expression was associated with the prognosis of different cancers in TCGA-TARGET.**

| **Overall survival** | | | | | | |
| --- | --- | --- | --- | --- | --- | --- |
| CancerCode | Case | pvalue | HR | Lower | Upper | Hazard Ratio(95%CI) |
| TCGA-ACC | N=77 | 0.04 | 1.65 | 1.02 | 2.66 | 1.65(1.02,2.66) |
| TCGA-BLCA | N=398 | 0.15 | 1.16 | 0.95 | 1.41 | 1.16(0.95,1.41) |
| TCGA-BRCA | N=1044 | 0.02 | 1.31 | 1.04 | 1.64 | 1.31(1.04,1.64) |
| TCGA-CESC | N=273 | 0.12 | 1.31 | 0.94 | 1.83 | 1.31(0.94,1.83) |
| TCGA-CHOL | N=33 | 0.73 | 1.14 | 0.54 | 2.42 | 1.14(0.54,2.42) |
| TCGA-COAD | N=278 | 0.13 | 0.70 | 0.44 | 1.11 | 0.70(0.44,1.11) |
| TCGA-COADREAD | N=368 | 2.10E-03 | 0.59 | 0.42 | 0.82 | 0.59(0.42,0.82) |
| TCGA-DLBC | N=44 | 0.71 | 0.84 | 0.34 | 2.06 | 0.84(0.34,2.06) |
| TCGA-ESCA | N=175 | 0.71 | 0.92 | 0.60 | 1.42 | 0.92(0.60,1.42) |
| TCGA-GBM | N=144 | 0.36 | 1.16 | 0.84 | 1.59 | 1.16(0.84,1.59) |
| TCGA-GBMLGG | N=619 | 1.00E-05 | 1.87 | 1.41 | 2.46 | 1.87(1.41,2.46) |
| TCGA-HNSC | N=509 | 0.03 | 1.30 | 1.02 | 1.66 | 1.30(1.02,1.66) |
| TCGA-KICH | N=64 | 0.13 | 2.18 | 0.79 | 6.05 | 2.18(0.79,6.05) |
| TCGA-KIPAN | N=855 | 0.03 | 0.82 | 0.69 | 0.98 | 0.82(0.69,0.98) |
| TCGA-KIRC | N=515 | 1.40E-04 | 0.68 | 0.56 | 0.83 | 0.68(0.56,0.83) |
| TCGA-KIRP | N=276 | 0.12 | 1.41 | 0.92 | 2.16 | 1.41(0.92,2.16) |
| TCGA-LAML | N=209 | 2.50E-03 | 1.38 | 1.12 | 1.70 | 1.38(1.12,1.70) |
| TCGA-LGG | N=474 | 5.30E-05 | 2.36 | 1.56 | 3.58 | 2.36(1.56,3.58) |
| TCGA-LIHC | N=341 | 8.10E-03 | 1.47 | 1.10 | 1.95 | 1.47(1.10,1.95) |
| TCGA-LUAD | N=490 | 0.55 | 1.07 | 0.85 | 1.34 | 1.07(0.85,1.34) |
| TCGA-LUSC | N=468 | 0.6 | 1.06 | 0.84 | 1.35 | 1.06(0.84,1.35) |
| TCGA-MESO | N=84 | 0.01 | 1.68 | 1.12 | 2.50 | 1.68(1.12,2.50) |
| TCGA-OV | N=407 | 0.66 | 0.96 | 0.78 | 1.17 | 0.96(0.78,1.17) |
| TCGA-PAAD | N=172 | 2.40E-03 | 1.78 | 1.23 | 2.59 | 1.78(1.23,2.59) |
| TCGA-PCPG | N=170 | 0.55 | 1.37 | 0.48 | 3.88 | 1.37(0.48,3.88) |
| TCGA-PRAD | N=492 | 0.49 | 1.35 | 0.57 | 3.21 | 1.35(0.57,3.21) |
| TCGA-READ | N=90 | 2.90E-03 | 0.48 | 0.29 | 0.79 | 0.48(0.29,0.79) |
| TCGA-SARC | N=254 | 0.34 | 1.15 | 0.87 | 1.52 | 1.15(0.87,1.52) |
| TCGA-SKCM | N=444 | 0.4 | 1.10 | 0.88 | 1.37 | 1.10(0.88,1.37) |
| TCGA-SKCM-M | N=347 | 0.17 | 1.19 | 0.93 | 1.53 | 1.19(0.93,1.53) |
| TCGA-SKCM-P | N=97 | 0.64 | 1.12 | 0.70 | 1.77 | 1.12(0.70,1.77) |
| TCGA-STAD | N=372 | 0.74 | 1.05 | 0.78 | 1.41 | 1.05(0.78,1.41) |
| TCGA-STES | N=547 | 0.63 | 1.06 | 0.84 | 1.33 | 1.06(0.84,1.33) |
| TCGA-TGCT | N=128 | 0.71 | 1.36 | 0.26 | 7.18 | 1.36(0.26,7.18) |
| TCGA-THCA | N=501 | 0.26 | 1.67 | 0.68 | 4.10 | 1.67(0.68,4.10) |
| TCGA-THYM | N=117 | 0.95 | 0.97 | 0.34 | 2.76 | 0.97(0.34,2.76) |
| TCGA-UCEC | N=166 | 0.17 | 0.78 | 0.55 | 1.11 | 0.78(0.55,1.11) |
| TCGA-UCS | N=55 | 0.53 | 1.20 | 0.67 | 2.15 | 1.20(0.67,2.15) |
| TCGA-UVM | N=74 | 0.07 | 1.94 | 0.95 | 3.97 | 1.94(0.95,3.97) |
| TARGET-LAML | N=142 | 0.02 | 1.33 | 1.06 | 1.67 | 1.33(1.06,1.67) |
| TARGET-ALL | N=86 | 0.84 | 0.97 | 0.70 | 1.34 | 0.97(0.70,1.34) |
| TARGET-ALL-R | N=99 | 0.77 | 1.04 | 0.78 | 1.40 | 1.04(0.78,1.40) |
| TARGET-NB | N=151 | 0.27 | 1.34 | 0.80 | 2.24 | 1.34(0.80,2.24) |
| TARGET-WT | N=80 | 0.26 | 1.27 | 0.84 | 1.92 | 1.27(0.84,1.92) |
| **Disease-specific survival** | | | | | | |
| CancerCode | Case | pvalue | HR | Lower | Upper | Hazard Ratio(95%CI) |
| TCGA-ACC | N=75 | 0.03 | 1.71 | 1.04 | 2.81 | 1.71(1.04,2.81) |
| TCGA-BLCA | N=385 | 0.21 | 1.16 | 0.92 | 1.48 | 1.16(0.92,1.48) |
| TCGA-BRCA | N=1025 | 0.84 | 1.03 | 0.77 | 1.38 | 1.03(0.77,1.38) |
| TCGA-CESC | N=269 | 0.18 | 1.30 | 0.89 | 1.88 | 1.30(0.89,1.88) |
| TCGA-CHOL | N=32 | 0.73 | 1.15 | 0.53 | 2.49 | 1.15(0.53,2.49) |
| TCGA-COAD | N=263 | 0.53 | 0.81 | 0.42 | 1.56 | 0.81(0.42,1.56) |
| TCGA-COADREAD | N=347 | 0.29 | 0.73 | 0.41 | 1.30 | 0.73(0.41,1.30) |
| TCGA-DLBC | N=44 | 0.44 | 0.63 | 0.19 | 2.04 | 0.63(0.19,2.04) |
| TCGA-ESCA | N=173 | 0.97 | 1.01 | 0.60 | 1.70 | 1.01(0.60,1.70) |
| TCGA-GBM | N=131 | 0.27 | 1.21 | 0.86 | 1.70 | 1.21(0.86,1.70) |
| TCGA-GBMLGG | N=598 | 1.60E-05 | 1.91 | 1.42 | 2.56 | 1.91(1.42,2.56) |
| TCGA-HNSC | N=485 | 0.35 | 1.16 | 0.85 | 1.57 | 1.16(0.85,1.57) |
| TCGA-KICH | N=64 | 0.03 | 4.22 | 1.12 | 15.97 | 4.22(1.12,15.97) |
| TCGA-KIPAN | N=840 | 0.02 | 0.77 | 0.62 | 0.96 | 0.77(0.62,0.96) |
| TCGA-KIRC | N=504 | 9.80E-06 | 0.59 | 0.47 | 0.75 | 0.59(0.47,0.75) |
| TCGA-KIRP | N=272 | 0.06 | 1.67 | 0.98 | 2.86 | 1.67(0.98,2.86) |
| TCGA-LGG | N=466 | 4.60E-05 | 2.49 | 1.61 | 3.86 | 2.49(1.61,3.86) |
| TCGA-LIHC | N=333 | 0.02 | 1.53 | 1.06 | 2.19 | 1.53(1.06,2.19) |
| TCGA-LUAD | N=457 | 0.76 | 1.05 | 0.79 | 1.39 | 1.05(0.79,1.39) |
| TCGA-LUSC | N=418 | 0.13 | 1.32 | 0.92 | 1.90 | 1.32(0.92,1.90) |
| TCGA-MESO | N=64 | 0.05 | 1.65 | 1.00 | 2.70 | 1.65(1.00,2.70) |
| TCGA-OV | N=378 | 0.4 | 0.91 | 0.73 | 1.13 | 0.91(0.73,1.13) |
| TCGA-PAAD | N=166 | 2.20E-03 | 1.96 | 1.27 | 3.02 | 1.96(1.27,3.02) |
| TCGA-PCPG | N=170 | 0.8 | 1.16 | 0.36 | 3.78 | 1.16(0.36,3.78) |
| TCGA-PRAD | N=490 | 0.24 | 2.33 | 0.55 | 9.85 | 2.33(0.55,9.85) |
| TCGA-READ | N=84 | 0.35 | 0.51 | 0.13 | 2.01 | 0.51(0.13,2.01) |
| TCGA-SARC | N=248 | 0.41 | 1.14 | 0.84 | 1.55 | 1.14(0.84,1.55) |
| TCGA-SKCM | N=438 | 0.36 | 1.12 | 0.88 | 1.42 | 1.12(0.88,1.42) |
| TCGA-SKCM-M | N=341 | 0.32 | 1.14 | 0.88 | 1.48 | 1.14(0.88,1.48) |
| TCGA-SKCM-P | N=97 | 0.33 | 1.33 | 0.74 | 2.38 | 1.33(0.74,2.38) |
| TCGA-STAD | N=351 | 0.59 | 1.11 | 0.76 | 1.62 | 1.11(0.76,1.62) |
| TCGA-STES | N=524 | 0.32 | 1.16 | 0.87 | 1.54 | 1.16(0.87,1.54) |
| TCGA-TGCT | N=128 | 0.7 | 0.69 | 0.11 | 4.49 | 0.69(0.11,4.49) |
| TCGA-THCA | N=495 | 0.92 | 1.07 | 0.31 | 3.63 | 1.07(0.31,3.63) |
| TCGA-THYM | N=117 | 0.97 | 0.97 | 0.22 | 4.23 | 0.97(0.22,4.23) |
| TCGA-UCEC | N=164 | 0.25 | 0.78 | 0.51 | 1.20 | 0.78(0.51,1.20) |
| TCGA-UCS | N=53 | 0.28 | 1.40 | 0.76 | 2.59 | 1.40(0.76,2.59) |
| TCGA-UVM | N=74 | 0.03 | 2.37 | 1.10 | 5.08 | 2.37(1.10,5.08) |
| **Disease-free interval** | | | | | | |
| CancerCode | Case | pvalue | HR | Lower | Upper | Hazard Ratio(95%CI) |
| TCGA-ACC | N=44 | 0.37 | 1.34 | 0.71 | 2.54 | 1.34(0.71,2.54) |
| TCGA-BLCA | N=184 | 0.91 | 1.03 | 0.67 | 1.57 | 1.03(0.67,1.57) |
| TCGA-BRCA | N=904 | 0.71 | 0.94 | 0.70 | 1.27 | 0.94(0.70,1.27) |
| TCGA-CESC | N=171 | 0.09 | 1.63 | 0.94 | 2.83 | 1.63(0.94,2.83) |
| TCGA-CHOL | N=23 | 0.51 | 1.40 | 0.52 | 3.74 | 1.40(0.52,3.74) |
| TCGA-COAD | N=103 | 0.38 | 1.62 | 0.55 | 4.74 | 1.62(0.55,4.74) |
| TCGA-COADREAD | N=132 | 0.83 | 1.10 | 0.45 | 2.71 | 1.10(0.45,2.71) |
| TCGA-DLBC | N=26 | 0.84 | 0.78 | 0.07 | 8.68 | 0.78(0.07,8.68) |
| TCGA-ESCA | N=84 | 0.67 | 0.84 | 0.37 | 1.90 | 0.84(0.37,1.90) |
| TCGA-GBMLGG | N=127 | 0.85 | 0.92 | 0.41 | 2.08 | 0.92(0.41,2.08) |
| TCGA-HNSC | N=128 | 0.53 | 0.81 | 0.43 | 1.55 | 0.81(0.43,1.55) |
| TCGA-KICH | N=29 | 0.61 | 1.58 | 0.27 | 9.08 | 1.58(0.27,9.08) |
| TCGA-KIPAN | N=319 | 0.25 | 1.30 | 0.83 | 2.03 | 1.30(0.83,2.03) |
| TCGA-KIRC | N=113 | 0.49 | 1.37 | 0.56 | 3.32 | 1.37(0.56,3.32) |
| TCGA-KIRP | N=177 | 0.18 | 1.44 | 0.84 | 2.48 | 1.44(0.84,2.48) |
| TCGA-LGG | N=126 | 0.68 | 0.84 | 0.37 | 1.91 | 0.84(0.37,1.91) |
| TCGA-LIHC | N=294 | 0.04 | 1.31 | 1.01 | 1.71 | 1.31(1.01,1.71) |
| TCGA-LUAD | N=295 | 0.94 | 1.01 | 0.72 | 1.43 | 1.01(0.72,1.43) |
| TCGA-LUSC | N=292 | 0.29 | 1.27 | 0.82 | 1.96 | 1.27(0.82,1.96) |
| TCGA-MESO | N=14 | 0.96 | 1.03 | 0.35 | 3.06 | 1.03(0.35,3.06) |
| TCGA-OV | N=203 | 0.56 | 0.92 | 0.69 | 1.22 | 0.92(0.69,1.22) |
| TCGA-PAAD | N=68 | 0.02 | 3.36 | 1.24 | 9.10 | 3.36(1.24,9.10) |
| TCGA-PCPG | N=152 | 0.07 | 8.68 | 0.81 | 93.28 | 8.68(0.81,93.28) |
| TCGA-PRAD | N=337 | 0.98 | 1.01 | 0.62 | 1.63 | 1.01(0.62,1.63) |
| TCGA-READ | N=29 | 0.22 | 0.32 | 0.05 | 2.05 | 0.32(0.05,2.05) |
| TCGA-SARC | N=149 | 0.89 | 1.02 | 0.73 | 1.43 | 1.02(0.73,1.43) |
| TCGA-STAD | N=232 | 0.58 | 0.86 | 0.50 | 1.46 | 0.86(0.50,1.46) |
| TCGA-STES | N=316 | 0.91 | 1.02 | 0.68 | 1.54 | 1.02(0.68,1.54) |
| TCGA-TGCT | N=101 | 0.36 | 1.34 | 0.72 | 2.49 | 1.34(0.72,2.49) |
| TCGA-THCA | N=352 | 0.52 | 1.24 | 0.64 | 2.42 | 1.24(0.64,2.42) |
| TCGA-UCEC | N=115 | 0.38 | 1.32 | 0.71 | 2.48 | 1.32(0.71,2.48) |
| TCGA-UCS | N=26 | 0.73 | 0.84 | 0.31 | 2.25 | 0.84(0.31,2.25) |
| **Progression-free interval** | | | | | | |
| **CancerCode** | **Case** | **pvalue** | **HR** | **Lower** | **Upper** | **Hazard Ratio(95%CI)** |
| TCGA-ACC | N=76 | 1.70E-03 | 1.93 | 1.27 | 2.93 | 1.93(1.27,2.93) |
| TCGA-BLCA | N=397 | 0.05 | 1.22 | 1.00 | 1.48 | 1.22(1.00,1.48) |
| TCGA-BRCA | N=1043 | 0.49 | 1.08 | 0.86 | 1.36 | 1.08(0.86,1.36) |
| TCGA-CESC | N=273 | 0.07 | 1.40 | 0.98 | 2.00 | 1.40(0.98,2.00) |
| TCGA-CHOL | N=33 | 0.79 | 1.11 | 0.52 | 2.38 | 1.11(0.52,2.38) |
| TCGA-COAD | N=275 | 0.95 | 0.99 | 0.64 | 1.52 | 0.99(0.64,1.52) |
| TCGA-COADREAD | N=363 | 0.49 | 0.89 | 0.63 | 1.25 | 0.89(0.63,1.25) |
| TCGA-DLBC | N=43 | 0.37 | 1.49 | 0.62 | 3.57 | 1.49(0.62,3.57) |
| TCGA-ESCA | N=173 | 0.72 | 1.08 | 0.71 | 1.63 | 1.08(0.71,1.63) |
| TCGA-GBM | N=143 | 0.76 | 0.95 | 0.69 | 1.32 | 0.95(0.69,1.32) |
| TCGA-GBMLGG | N=616 | 5.70E-05 | 1.62 | 1.28 | 2.06 | 1.62(1.28,2.06) |
| TCGA-HNSC | N=508 | 0.74 | 1.04 | 0.82 | 1.32 | 1.04(0.82,1.32) |
| TCGA-KICH | N=64 | 0.29 | 1.58 | 0.68 | 3.72 | 1.58(0.68,3.72) |
| TCGA-KIPAN | N=845 | 0.04 | 0.83 | 0.70 | 0.99 | 0.83(0.70,0.99) |
| TCGA-KIRC | N=508 | 1.10E-04 | 0.67 | 0.55 | 0.82 | 0.67(0.55,0.82) |
| TCGA-KIRP | N=273 | 0.15 | 1.32 | 0.91 | 1.91 | 1.32(0.91,1.91) |
| TCGA-LGG | N=472 | 2.10E-03 | 1.62 | 1.19 | 2.21 | 1.62(1.19,2.21) |
| TCGA-LIHC | N=340 | 0.02 | 1.33 | 1.05 | 1.67 | 1.33(1.05,1.67) |
| TCGA-LUAD | N=486 | 0.89 | 1.02 | 0.82 | 1.26 | 1.02(0.82,1.26) |
| TCGA-LUSC | N=467 | 0.18 | 1.21 | 0.92 | 1.59 | 1.21(0.92,1.59) |
| TCGA-MESO | N=82 | 0.62 | 1.12 | 0.72 | 1.72 | 1.12(0.72,1.72) |
| TCGA-OV | N=407 | 0.44 | 0.93 | 0.78 | 1.12 | 0.93(0.78,1.12) |
| TCGA-PAAD | N=171 | 2.80E-03 | 1.73 | 1.21 | 2.48 | 1.73(1.21,2.48) |
| TCGA-PCPG | N=168 | 0.34 | 1.41 | 0.69 | 2.89 | 1.41(0.69,2.89) |
| TCGA-PRAD | N=492 | 0.68 | 1.06 | 0.80 | 1.41 | 1.06(0.80,1.41) |
| TCGA-READ | N=88 | 0.23 | 0.71 | 0.41 | 1.24 | 0.71(0.41,1.24) |
| TCGA-SARC | N=250 | 0.61 | 1.07 | 0.83 | 1.36 | 1.07(0.83,1.36) |
| TCGA-SKCM | N=434 | 0.78 | 0.97 | 0.82 | 1.16 | 0.97(0.82,1.16) |
| TCGA-SKCM-M | N=338 | 0.46 | 0.93 | 0.76 | 1.13 | 0.93(0.76,1.13) |
| TCGA-SKCM-P | N=96 | 0.17 | 1.37 | 0.88 | 2.14 | 1.37(0.88,2.14) |
| TCGA-STAD | N=375 | 0.84 | 1.03 | 0.76 | 1.40 | 1.03(0.76,1.40) |
| TCGA-STES | N=548 | 0.12 | 1.20 | 0.95 | 1.51 | 1.20(0.95,1.51) |
| TCGA-TGCT | N=126 | 0.72 | 1.10 | 0.65 | 1.88 | 1.10(0.65,1.88) |
| TCGA-THCA | N=499 | 0.84 | 1.05 | 0.67 | 1.65 | 1.05(0.67,1.65) |
| TCGA-THYM | N=117 | 0.35 | 1.42 | 0.69 | 2.92 | 1.42(0.69,2.92) |
| TCGA-UCEC | N=166 | 0.54 | 0.90 | 0.64 | 1.26 | 0.90(0.64,1.26) |
| TCGA-UCS | N=55 | 0.26 | 1.37 | 0.79 | 2.37 | 1.37(0.79,2.37) |
| TCGA-UVM | N=73 | 4.60E-04 | 3.50 | 1.74 | 7.06 | 3.50(1.74,7.06) |

**Table S2 GSPT1/ERF3a gene expression was associated with the prognosis of different cancers in PrognoScan.**

| **DATASET** | **CANCER TYPE(SUBTYPE)** | **ENDPOINT** | **N** | **COX P-VALUE** | **HR [95% CI^low^ - CI^upp^]** |
| --- | --- | --- | --- | --- | --- |
| GSE2658 | Blood cancer  (MM) | DSS | 559 | 0.017735 | 1.95 [1.12 - 3.38] |
| GSE5122 | Blood cancer  (AML) | OS | 58 | 0.040626 | 0.48 [0.23 - 0.97] |
| GSE12417  -GPL96 | Blood cancer  (AML) | OS | 163 | 0.004187 | 0.66 [0.50 - 0.88] |
| GSE4271  -GPL96 | Brain cancer (Astrocytoma) | OS | 77 | 0.03707 | 2.42 [1.05 - 5.55] |
| GSE12276 | Breast cancer | RFS | 204 | 0.014285 | 1.56 [1.09 - 2.22] |
| GSE1456  -GPL96 | Breast cancer | RFS | 159 | 0.002913 | 3.12 [1.47 - 6.59] |
| GSE1456  -GPL96 | Breast cancer | DSS | 159 | 0.005777 | 3.41 [1.43 - 8.13] |
| GSE14333 | Colorectal cancer | DFS | 226 | 0.015825 | 0.39 [0.18 - 0.84] |
| GSE22138 | Eye cancer (Uveal melanoma) | DMFS | 63 | 0.046649 | 1.39 [1.00 - 1.94] |
| GSE13213 | Lung cancer (Adenocarcinoma) | OS | 117 | 0.000859 | 1.60 [1.21 - 2.11] |
| GSE4573 | Lung cancer (Squamous cell carcinoma) | OS | 129 | 0.042309 | 2.58 [1.03 - 6.42] |
| GSE31210 | Lung cancer (Adenocarcinoma) | OS | 204 | 0.002942 | 0.38 [0.20 - 0.72] |
| GSE31210 | Lung cancer (Adenocarcinoma) | RFS | 204 | 0.000277 | 0.41 [0.25 - 0.66] |
| GSE14764 | Ovarian cancer | OS | 80 | 0.001278 | 4.57[1.81 - 11.5] |
| GSE19234 | Skin cancer (Melanoma) | OS | 38 | 0.013242 | 3.95 [1.33- 11.69] |

Annotation: OS, overall survival; RFS, relapse free survival; DSS, disease specific survival; DFS, Disease Free Survival; DMFS, Distant Metastasis Free Survival; DRFS, Distant Recurrence Free Survival.

**Table S3 Abbreviation.**

| **Abbreviation** | **Full Term** |
| --- | --- |
| ADC | Antibody-drug conjugate |
| ALL | Acute lymphoblastic leukemia |
| AML | Acute myeloid leukemia |
| BRCA | Breast cancer |
| BTK | Bruton Tyrosine Kinase |
| C domain | C-terminal domain |
| CNV | Copy number variation |
| COAD | Colon Adenocarcinoma |
| CRBN | Cereblon |
| CRC | Colorectal cancer |
| CRPC | Castration-resistant prostate cancer |
| DACs | Degrader antibody conjugates |
| DLBC | Diffuse Large B-cell Lymphoma |
| DLBCL | Diffuse large B-cell lymphoma |
| ER | Endoplasmic reticulum |
| eRF3a | Eukaryotic release factor 3a |
| ESCA | Esophageal Carcinoma |
| GBM | Glioblastoma |
| GC | Gastric cancer |
| GSPT1 | G1 to S phase transition protein |
| GTP | Guanosine triphosphate |
| HER2 | Human epidermal growth factor receptor |
| HNSC | Head and Neck Squamous Cell Carcinoma |
| IAP | Inhibitor of apoptosis protein |
| ISR | Integrated stress response |
| LIHC | Liver Hepatocellular Carcinoma |
| lncRNA | Long non-coding RNA |
| LGG | Low-Grade Glioma |
| LUAD | Lung Adenocarcinoma |
| MGD | Molecular glue degrader |
| miRNA | MicroRNA |
| mTOR | Mammalian TOR |
| N domain | N-terminal domain |
| NHL | Non-hodgkin lymphoma |
| NMD | Nonsense-mediated mRNA decay |
| NSCLC | Non-small cell lung cancer |
| OV | Ovarian Cancer |
| PABP | Poly(A)-binding protein |
| PPI | Protein-Protein Interaction |
| PRAD | Prostate cancer |
| PROTAC | Proteolysis-targeting chimera |
| PTC | Premature stop codon |
| PTM | Protein translational modification |
| R/R | Relapsed/refractory |
| STAD | Stomach Adenocarcinoma |
| TCGA | The Cancer Genome Atlas |
| TGCT | Testicular Germ Cell Tumor |
| TMB | Tumor mutation burden |
| TPD | Targeted protein degradation |
| UCEC | Uterine Corpus Endometrial Carcinoma |
| UVM | Uveal Melanoma |
| VEN | Venetoclax |
